# Chromosome-level genome assembly of *Cornus officinalis* reveals the evolution of loganin biosynthesis

**DOI:** 10.1093/hr/uhaf259

**Published:** 2025-09-24

**Authors:** Xiang Zhang, Jiangbo Xie, Jiadong Wu, Haoyu Zhang, Zhelun Jin, Qing Liu, Deqiang Zhang

**Affiliations:** State Key Laboratory of Tree Genetics and Breeding, National Engineering Research Center of Tree Breeding and Ecological Restoration, Key Laboratory of Genetics and Breeding in Forest Trees and Ornamental Plants, Ministry of Education, College of Biological Sciences and Biotechnology, Beijing Forestry University, Beijing 100083, China; State Key Laboratory of Tree Genetics and Breeding, National Engineering Research Center of Tree Breeding and Ecological Restoration, Key Laboratory of Genetics and Breeding in Forest Trees and Ornamental Plants, Ministry of Education, College of Biological Sciences and Biotechnology, Beijing Forestry University, Beijing 100083, China; State Key Laboratory of Tree Genetics and Breeding, National Engineering Research Center of Tree Breeding and Ecological Restoration, Key Laboratory of Genetics and Breeding in Forest Trees and Ornamental Plants, Ministry of Education, College of Biological Sciences and Biotechnology, Beijing Forestry University, Beijing 100083, China; State Key Laboratory of Tree Genetics and Breeding, National Engineering Research Center of Tree Breeding and Ecological Restoration, Key Laboratory of Genetics and Breeding in Forest Trees and Ornamental Plants, Ministry of Education, College of Biological Sciences and Biotechnology, Beijing Forestry University, Beijing 100083, China; State Key Laboratory of Tree Genetics and Breeding, National Engineering Research Center of Tree Breeding and Ecological Restoration, Key Laboratory of Genetics and Breeding in Forest Trees and Ornamental Plants, Ministry of Education, College of Biological Sciences and Biotechnology, Beijing Forestry University, Beijing 100083, China; CSIRO Agriculture and Food, Black Mountain, Australian Capita Territory, Canberra, ACT, 2601， Australia; State Key Laboratory of Tree Genetics and Breeding, National Engineering Research Center of Tree Breeding and Ecological Restoration, Key Laboratory of Genetics and Breeding in Forest Trees and Ornamental Plants, Ministry of Education, College of Biological Sciences and Biotechnology, Beijing Forestry University, Beijing 100083, China; College of Horticulture and Landscape Architecture, Beijing Vocational College of Agriculture, Beijing 102442, China

## Abstract

*Cornus officinalis* is a traditional medicinal plant known for producing loganin, a bioactive iridoid glycoside with potential anticancer properties. However, the absence of a high-quality reference genome has limited insights into its biosynthetic pathways. Here, we present a chromosome-level genome assembly of *C. officinalis* with a size of 2.85 Gb. Comparative genomic analysis revealed that the genome expansion and longer gene structures, relative to other *Cornales* species, are primarily due to a recent expansion of transposable elements. In this study, we identified unique biosynthetic gene clusters coding multiple core enzymes, including loganin acid O-methyltransferase (LAMT), secologanin synthase (SLS), and cytochrome P450, all of which catalyze sequential steps leading to loganin formation. LAMT enzymes from *C. officinalis* capable of catalyzing the C-9 hydroxylation of loganin acid were identified, whereas the homolog (CoLMAT) was not found to possess this activity. Additionally, molecular docking studies revealed critical residues in CoLAMT that govern substrate positioning, providing insights into the mechanism of C-9 regioselective hydroxylation. Further characterization of 7-deoxyloganicacid hydroxylase, LAMT, and SLS enzymes allowed us to elucidate the complete biosynthetic pathway of major loganin derivatives in the medicinal plant *C. officinalis*. Finally, we introduced CoLAMT and its upstream genes into *Nicotiana benthamiana* and successfully achieved the *de novo* biosynthesis of a series of loganin derivatives. This work reveals key evolutionary and molecular mechanisms in loganin biosynthesis, providing insights into biotechnological applications in anticancer drug development.

## Introduction

*Cornus officinalis,* a medicinally and economically important member of the *Cornaceae* family, is native to China and widely distributed across 13 provinces at elevations ranging from 400 to 1500 m above sea level [1, 2]. Demonstrating exceptional adaptability, this species has been successfully introduced to warm temperate and subtropical regions across southern Europe, eastern Asia, and eastern North America [3, 4]. The fruit of *C. officinalis* has long been used in traditional Chinese medicine to treat spleen deficiency, nourish yin, and reduce spontaneous perspiration [5, 6]. As a key ingredient in several commercial formulations, including Liuwei Dihuang Wan, it contributes substantially to a market valued in the hundreds of millions of dollars annually [7–9].

The medicinal value of *C. officinalis* is primarily attributed to terpenoid compounds, which have been widely studied for their multiple effects, including antitumor activity, blood glucose-lowering, and antioxidant properties [10–13]. Terpenoid compounds are important plant secondary metabolites synthesized through the methylerythritol phosphate (MEP) and mevalonate (MVA) pathways [2, 8, 14, 15]. The main terpenoid components of *C. officinalis* include loganin, morroniside, ursolic acid, and oleanolic acid [16, 17]. Among these, loganin stands out for its potent anticancer effects. Modern pharmacological research has shown that loganin has significant effects on cancer, diabetes, and kidney diseases [18–20]. Recent studies have highlighted that loganin can bind to topoisomerase I and prevent the re-ligation of DNA strands, effectively inhibiting the proliferation of tumor cells [18, 19, 21]. Previous studies have indicated that loganin may also serve as a potential adjunctive therapeutic approach for non-alcoholic fatty liver disease (NAFLD) by modulating gut microbiota and lipid metabolism [22]. Therefore, loganin (including its derivatives) is widely used in the treatment of malignant tumors and NAFLD. Despite its significant economic value, the biosynthetic pathway of loganin remains unclear, and its low yield limits industrial production.

Loganin biosynthesis entails a series of enzymatic modifications, including hydroxylation, methylation, and glycosylation. The biosynthetic pathway begins with the condensation of isoprenyl diphosphate and dimethylallyl diphosphate to form geranylgeranyl diphosphate (GGPP), which is cyclized by the plastid-localized enzyme geraniol synthase (GES) to yield geraniol [23–25]. Geraniol subsequently undergoes multiple enzymatic modifications, including oxidation, reduction, methylation, and glycosylation, to produce loganic acid [26, 27]. In the final step, loganic acid is O-methylated by LAMT to yield loganin [28, 29]. Although more than 16 enzymes involved in the biosynthesis of loganin have been identified, the pathway remains highly complex, and several key steps, such as C8 oxygenation and C9 hydroxylation, remain to be fully clarified. CaLAMT has been explored for the treatment of cancer and has been approved by the US Food and Drug Administration (FDA) [30]. Notably, C-9 hydroxylated LAMT analogues have shown higher potency than the anticancer drug CaLAMT in liver cancer (HepG2), ovarian cancer (A2780), and colon cancer (SW480) cell lines. In ovarian cancer (A2780) cell lines, their activity is nearly six times greater than that of CaLAMT [31–36]. Nevertheless, the absence of a comprehensive genome sequence for *C. officinalis* continues to impede the application of C-9 hydroxylated LAMT derivatives’ applicability for developing new anticancer drugs.

Advances in genome sequencing, assembly technologies, and supporting bioinformatics tools have injected new vitality into the development of genomic and molecular biological research on medicinal plants, greatly facilitating the construction of high-quality genomes for these plants [28, 37–40]. In line with this progress, the ‘Herbal Medicinal Plant Genome Project’ has been proposed, aiming to conduct whole-genome sequencing and post-genomics research on medicinal plants of significant economic value [41]. Despite its broad genetic diversity, the chromosome number of *C. officinalis* remains unresolved at the genomic level. Therefore, decoding the complete genome of *C. officinalis* is crucial for elucidating the biosynthetic pathway of loganin. In this study, we report the assembly of a high-quality *C. officinalis* genome to bridge the existing knowledge gap, coupled with a comprehensive genomic analysis aimed at deciphering the adaptive evolution and functional innovations within the *Cornaceae* family. Additionally, molecular docking studies further revealed critical residues in *CoLAMT* that govern substrate positioning, providing insights into the mechanism of C-9 regioselective hydroxylation. To further enhance the synthesis efficiency of cornuside and optimize its biosynthetic pathway, metabolic engineering techniques will be required in the near future. Collectively, our findings not only deepen our understanding of loganin biosynthetic pathways but also chart promising new avenues for advancements in synthetic biology and drug development.

**Figure 1:**
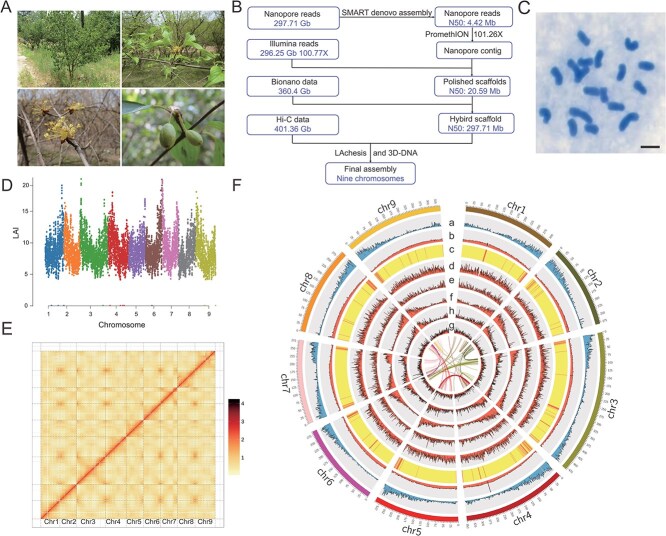
Genomic features of *Cornus officinalis*. **A** Morphologic characteristics of *C. officinalis*, including its tree structure, leaves, flowers, and fruits. **B** Genome assembly strategy used for *C. officinalis*. **C** Mitotic chromosome spread of *C. officinalis*, confirming its diploid genome with 2*n* = 18 chromosomes. Bar, 2 μm. **D** LAI (long terminal repeat assembly index) assessment for each assembled chromosome. The average LAI score is 11.6, indicating a reference-level genome assembly. LAI (LTR Assembly Index) score indicating the quality of the genome assembly in repeat-rich regions. An LAI score above 10 suggests reference-quality assembly. **E** Heatmap of *C. officinalis* nine pseudomolecules scaffolding with Hi-C data. **F** Circos plot illustrates various genomic features of *C. officinalis*. a, gene density per chromosome (Mb). b, GC content distribution. c, Repeat elements coverage. d, Density of *Gypsy* LTR retrotransposons (LTR-RTs). e, Density of *Copia* LTR-RTs. f, Density of LINE/L1. g, mRNA coverage. h, snRNA coverage. Center, curve lines link indicate syntenic blocks in the genome.

## Results

### High-quality genome assembly and annotation of *C. officinalis*

The genome of *C. officinalis* was sequenced and assembled using a multifaceted approach that integrated Illumina short-read sequencing (100.77x coverage) with Oxford Nanopore Technologies (ONT) (101.26x coverage), complemented by BioNano optical mapping and Hi-C sequencing (Fig. 1A and B). A highly contiguous *de novo* assembly was achieved by incorporating 297.71 Gb of Nanopore long reads alongside 296.25 Gb of Illumina whole-genome sequencing data. Leveraging Hi-C data, we successfully anchored 366 assembled scaffolds into nine pseudochromosomes (2*n* = 18), collectively covering 96.84% of the genome (Table S1). The finalized genome assembly spans 2.96 Gb, featuring an impressive scaffold N50 of 297.71 Mb (Fig. 1E and F and Table S2), a metric that aligns closely with genome size estimates derived from flow cytometry (2.8 Gb) and *K*-mer analysis (2.97 Gb) (Figs 1C and S1).

The completeness of the *C. officinalis* genome assembly and its annotated gene sets was vigorously assessed using the Benchmarking Universal Single-Copy Orthologs (BUSCO) approach [42]. The assembly achieved a remarkable completeness score of 98.80% (encompassing 2298 BUSCOs), while the annotated genes reached a completeness of 95.50% (2221 BUSCOs) (Table S3). Complementary quality assessments, based on the Illumina data mapping rate and sequence coverage, further highlighted the assembly’s integrity, with values of 99.65% and 97.57%, respectively. Additionally, transcriptomic analysis across diverse tissues, including young and mature leaves, fruits, seeds, stems, and roots, yielded a robust read-mapping rate of 93.00%. Notably, a long terminal repeat (LTR) assembly index (LAI) score of 11.6 confirmed that the assembly meets reference-level quality standards (Fig. 1D). Overall, these findings unequivocally demonstrate that the *C. officinalis* genome assembly is of exceptionally high quality.

A total of 41 911 protein-coding genes were predicted in the *C. officinalis* genome (Table S4). Among these, 40 037 genes (95.5%) were functionally annotated through similarity searches against several protein databases. Specifically, annotations were aligned to 39 713 (94.80%) genes using the NCBI non-redundant (NR) protein database, 31 526 (75.20%) *via* the Swiss-Prot protein database, 37 882 genes (90.40%) through the InterPro database, 29 923 genes (71.40%) the protein families database (Pfam), 21 759 genes (51.90%) using the Gene Ontology (GO) database, and 32 948 genes (78.60%) via the Kyoto Encyclopedia of Genes and Genomes (KEGG). The assembled genome also contains a substantial number of repetitive sequences, totaling 2077.44 Mb, accounting for 75.04% of the genome (Table S5). Within this repetitive landscape, LTR retrotransposons (LTR-RTs) are predominant, representing 59.93% of the genome, followed by DNA transposons (2.58%) and long interspersed nuclear elements (LINEs; 1.65%). Notably, the *Ty3/Gypsy* and *Ty1/Copia* families are the two major classes of LTRs, comprising 41.26% and 9.66% of the genome, respectively. This TE content is markedly higher than previously reported values, highlighting the extensive and complex repetitive nature of the *C. officinalis* genome.

### Expansion of *NAC* and *TPS* gene families related to environmental adaptability

**Figure 2:**
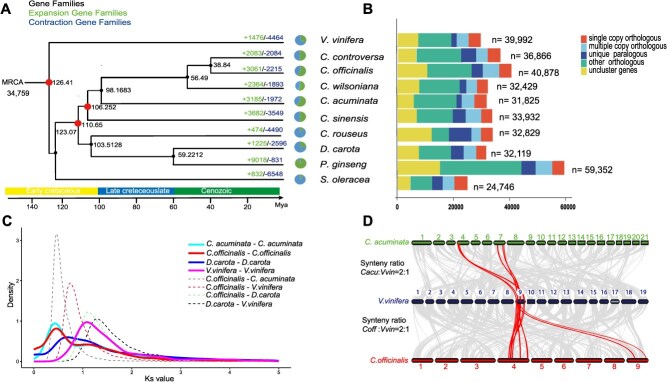
Phylogenetic analysis of the *Cornus officinalis* genome. **A** Phylogenetic tree depicting the evolutionary relationship between *C. officinalis* and seven other plant species. Fossil-calibrated molecular clock chronogram showing estimated divergence times among major families of *Cornales*. Node bars indicate 95% highest posterior density intervals. Bootstrap values for each node are 100. The colored branches indicate the major time periods (Early Cretaceous, Late Cretaceous, Cenozoic). The black number at each node denotes estimated divergence time, while red nodes indicate the known divergence times of monocots and dicots, *Apiales* and *Cornales*, and *C. officinalis* and *Camellia sinensis*, respectively. The diagram shows the number of different gene family copy variations among the analyzed plant species. Gene family expansions and contractions are indicated along the tree branches with the number of gene family expansions (green) and contractions (blue). **B** Stacked bar chart illustrating the classification of gene families across different datasets, with each bar segment representing a gene category: single copy orthologous, multiple copy orthologous, unique paralogous, other orthologous, and uncluster genes. The sample sizes (n) for each dataset are indicated on the right. **C**  *Ks* (synonymous substitution rate) distribution among homologous gene pairs within *C. officinalis*, *Camptotheca acuminata*, *Daucus carota*, and *Vitis vinifera*. Red line represents *C. officinalis*; light gray line represents *C. acuminata*; blue line represents *D. carota*; pink line represents *V. vinifera*. **D** Collinear relationship between *C. officinalis*, *C. acuminata*, and *V. vinifera* chromosomes. The collinearity pattern shows that ancestral genomic regions in *V. vinifera* genome can typically be traced to two homologous regions in *C. officinalis*, suggesting an additional genome duplication event. The chromosomes of *C. officinalis*, *V. vinifera*, and *C. acuminata* are represented by red, purple, and green boxes, respectively. Further information on gene-level syntenic depth is provided in Fig. S4.

Utilizing a dataset of 295 single-copy genes, we constructed a phylogenetic tree encompassing nine *Asterid* species (*C. officinalis*, *Camellia sinensis*, *Daucus carota*, *Camptotheca acuminata*, *C. controversa*, *C. wilsoniana*, *Catharanthus roseus*, *Panax ginseng,* and *Spinacia oleracea*), with *Vitis vinifera* designated as the outgroup (Fig. 2A, Fig. S2, Data S1). Molecular clock analysis indicates that *C. officinalis* diverged from *C. acuminata* (a representative of the *Cornales* group) approximately 98.16 million years ago (Mya), with the 95% confidence interval (CI) ranging from 89.3 to 107.4 Mya.

The analysis of synonymous substitution (*Ks*) values for paralogs in the genomes of *C. officinalis*, *C. acuminata*, *D. carota,* and *V. vinifera* revealed a pronounced peak in the range of *Ks* = 1.1–1.8 Mya (Fig. 2B). This peak is consistent with a well-documented whole genome duplication (WGD) event shared by core true dicotyledons [26, 43, 44]. Furthermore, the *Ks* distribution for paralogous genes between *C. officinalis* and *C. acuminata* exhibited a distinct peak at *Ks* = 0.40–0.45 Mya (Fig. 2B), highlighting a recent WGD event that is also evident in *C. acuminata* of the *Cornales* [31]. Synteny analysis further revealed an overall 2:1 syntenic relationship between *C. officinalis* and *V. vinifera,* in contrast to a clear 1:1 syntenic correspondence between *C. officinalis* and *C. acuminata* (Figs 2C and S3-S4). Collectively, these findings strongly suggest that *C. officinalis* has experienced a recent *Cornaceae* lineage-specific WGD [45, 46].

Comparative genomic analyses were conducted to examine the differential evolution and adaptation between low-altitude *C. officinalis* and the two high-altitude species, *C. controversa* and *C. wilsoniana*. A total of 3061 expanded genes were determined in *C. officinalis* relative to the two high-altitude species. Manual annotation of known transcription factor (TF) and transcription regulator (TR) families led to the identification of 2090 TFs and 445 TRs in *C. officinalis* (Data S2). Members of the *WAKY*, *GRF*, *NAC* TF families, as well as the TPS gene family, which are known to be involved in stress responses, were further investigated for their evolutionary significance in *C. officinalis*.

Particularly striking was the extensive expansion of the NAC subfamily Group IX in *C. officinalis*, which contained 69 genes, substantially higher than those observed in *C. controversa* and *C. wilsoniana* (24 and 16 genes, respectively; Fig. S5A and B). A detailed examination revealed that 6, 14, and 8 copies of *NAC* genes are clustered on chromosomes 2, 3, and 4, spanning 79, 122, and 94 kb, respectively (Fig. S5C and D). Analysis of gene family expression patterns reveals that *NAC12* highly expressed under stress conditions (Fig. S6). To further investigate the role of the *NAC12* family in stress responses, we evaluated the drought tolerance of three-month-old wild-type (WT) plants and 84 K (*P. alba × P. glandulosa*) *NAC12*-overexpressing (OE) lines (Fig. S7A). Following a 28-day drought treatment, leaves of WT plants displayed wilting symptoms, with a 50% reduction in leaf water content, while NAC12-OE lines showed no apparent symptoms, maintaining 64% of their original leaf water content. These results suggest that overexpression of NAC12 confers enhanced drought resistance, with NAC12-OE lines exhibiting a 28% higher leaf water retention compared to WT plants (Fig. S7B and Table S6). Additionally, NAC12-OE lines exhibited 2.5-fold higher CO_2_ assimilation and a 25% increase in growth rate compared to WT plants (Fig. S7C). Further biochemical analysis revealed additional stress-tolerance traits in NAC12-OE lines. Under drought stress, soluble sugar content in OE plants increased by 35% compared to WT plants (Fig. S7D), suggesting enhanced osmotic balance and reduced risk of dehydration. This increase in soluble sugar accumulation suggests that NAC12-OE lines are better equipped to maintain osmotic balance and avoid dehydration under water deficit. Malondialdehyde (MDA), a marker of oxidative stress, was significantly lower in NAC12-OE lines, suggesting reduced oxidative damage (Fig. S7F). Free proline levels were also markedly higher, showing a 50% increase over WT, indicating an improved capacity for osmotic adjustment and cellular protection (Fig. S7G). Collectively, these in water retention, photosynthetic performance, and growth strongly suggest that *NAC12* overexpression confers enhanced drought tolerance by promoting efficient water use and sustaining metabolic activity under water-limited conditions (Fig. S7). Such traits may have been positively selected during evolution, highlighting the potential role of NAC12 in adaptive responses to arid environments.

Terpenoid metabolism plays important roles in mediating environmental responses and disease resistance [47]. KEGG pathway analysis revealed that genes associated with the terpenoid metabolism pathway are significantly expanded in *C. officinalis* (*P* < 0.05; Fig. S8). Comparative genomic analysis further demonstrated a marked expansion of the *TPS* gene family in *C. officinalis,* which now comprises 88 genes, significantly more than the 39 and 32 genes observed in *Arabidopsis thaliana* and *C. acuminata*, respectively, with the *TPS*-a/b/g subfamilies exhibiting particularly notable growth (Fig. S9A and B). Chromosomal mapping of these *TPS* genes revealed a distinctive organization in tandem repeats, especially on chromosomes 3, 5, 7, and 8, where up to 10 *TPS* genes are clustered together (Fig. S9C). These findings underscore the role of gene family expansions, particularly for *NAC* and *TPS* genes, in enhancing *C. officinalis*’s ability to adapt to diverse and challenging environmental conditions.

### Reduced DNA methylation levels of transposable elements over evolutionary time

**Figure 3:**
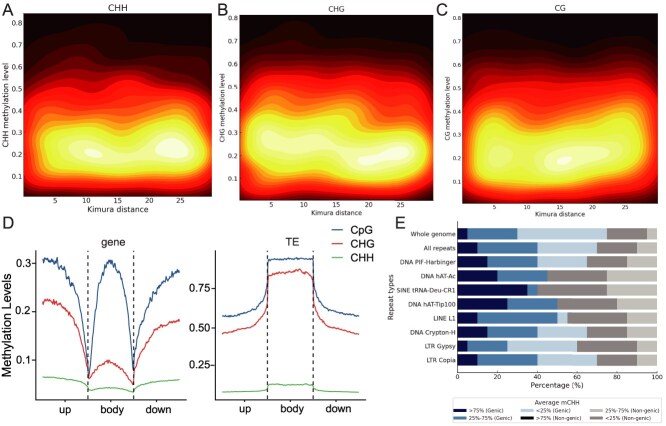
DNA methylation landscape of the *Cornus officinalis* genome. **A-C.** Heatmaps showing the relationship between methylation level and Kimura distance for transposable elements (TEs) in three cytosine methylation contexts: (A) CHH, (B) CHG, and (C) CG. The y-axis indicates the average methylation level, and the x-axis represents the Kimura substitution level, a proxy for TE age. Warmer indicate higher TE densities. In all contexts, younger TEs (lower Kimura distances) generally exhibit reduced methylation, particularly in CHH and CHG contexts, suggesting dynamic epigenetic regulation associated with TE age. Dots represent the correlation strength between methylation level and TE age. Older TEs exhibit lower methylation levels across all contexts, consistent with gradual epigenetic decay over evolutionary time. The R-values for plots A, B, and C are −0.164, −0.371, and −0.178, respectively. **D** Distribution of DNA methylation levels across different genomic regions, including genes and TEs. **E** Distribution of mCHH within representative repeat regions, analyzed in 100 bp sliding disjoint windows. The figure shows the distribution of mCHH levels across genomic and non-genomic contexts. For genomic contexts, the blocks indicate the proportions with mCHH >75%, 25–75%, and <25%. For non-genomic contexts, the blocks indicate the proportions with mCHH <25%, 25–75%, and >75%.

Recent studies have shown that newly duplicated genes tend to be hypermethylated compared to their older counterparts, with DNA methylation levels gradually declining over evolutionary time [20]. To investigate the relationship between DNA methylation and the age of TE insertion, we calculated Kimura distances [48] for all TE copies across various families. Our analysis revealed a distinct burst of TE insertions at a Kimura distance of approximately 20 (Fig. 3A–C). In the *C. officinalis* genome, the average methylation levels were 93.42% for mCG, 71.64% for mCHG, and 2.0% for mCHH (Fig. 3D). A negative correlation was observed between the age of TE insertion and DNA methylation levels across all three sequence contexts (*r* = −0.45). Furthermore, regions within genes that harbor transposable elements (TEs) exhibited higher methylation levels compared to non-TE-bearing regions (Fig. 3D and E) (*r* = 0.60). Exons are characterized by elevated mCG levels coupled with reduced mCHG and mCHH levels, a phenomenon referred to as gene body methylation (gbM) (*r* = 0.55). This pattern suggests that the evolution of gbM may be intricately linked to the silencing of TEs *via* DNA methylation in regions proximal to genes.

Additionally, our comparative analysis between leaves and fruits identified 2217 high-confidence differentially methylated genes (DMGs) across all methylation contexts (Fig. S10A). GO enrichment analysis of these DMGs showed significant differential methylation (*P* < 0.05) in pathways related to terpenoid backbone biosynthesis (35 genes), flavonoid biosynthesis (22 genes), and cell wall biosynthesis (17 genes) (Fig. S10B). Among the DMGs, 49 were implicated in terpenoid biosynthesis, including gene *Co.595.10*, which encodes the 7-deoxyloganic acid hydroxylase enzyme. These findings suggest that epigenetic modifications, such as DNA methylation, may modulate secondary metabolic pathways, such as loganin production, thereby influencing the plant’s adaptive responses to environmental challenges.

**Figure 4:**
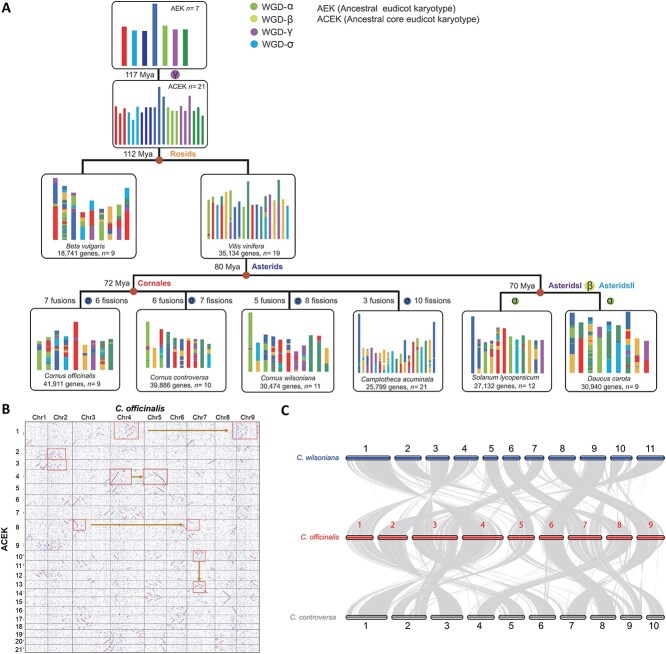
Polyploidization analysis of the *Cornus officinali*s genome. **A** Evolutionary reconstruction of chromosome fusions and fissions shaping modern *C. officinalis* karyotypes from the ancestral core eudicot karyotype (ACEK). The n = 7 ACEK is illustrated at the top, with a 21-colour code scheme representing the seven ancestral chromosomes. Modern genomes are shown below, with blocks indicating chromosomal origins from the protochromosomes. The top panel shows the ancestral eudicot karyotype (AEKs) at 117 Mya, with gene duplications represented by bars corresponding to WGD-α , WGD-β , WGD-γ , and WGD-δ events. The ACEK (112 Mya) is shown beneath, using the same whole-genome duplication (WGD) scheme. Evolutionary relationships of key taxa within the core eudicot clade are illustrated, with the rosids lineage including *Beta vulgaris* (9 samples) and *Vitis vinifera* (19 samples) compared based on gene content. The *Cornales* and *Asterids* lineages are shown with different numbers of gene fusions and fissions across species, including *Cornus officinalis* (9 samples), *Cornus controversa* (10 samples), *Cornus wilsoniana* (11 samples), *Camptotheca acuminata* (21 samples), *Solanum lycopersicum* (12 samples), and *Daucus carota* (9 samples). Gene counts and the number of fusion and fission events are indicated for each species. The protochromosome number of the most recent common ancestor and the chromosome number of extant species are indicated using Roman numerals from an up-to-down hierarchy. Each segment represents a distinct ancestral chromosome. **B** Syntenic block dot plot comparing *C. officinali*s with the ACEK. A 2:2 syntenic depth ratio is shown using squares and solid lines. **C** Collinear relationships between *Cornus controversa*, *C. officinali*s, and *Cornus wilsoniana*. The banded lines in the background indicate syntenic blocks spanning more than 15 genes, illustrating chromosomal conservation and structural variation among these closely related species.

### Ancestral karyotypes in modern *Cornales* driving rapid evolution of chromosome structure

Karyotype evolution encapsulates the chromosomal transformations that bridge the ancestral genome and its modern descendants. In this study, we reconstructed the evolutionary trajectory of karyotypes across eight species, including *Beta vulgaris* (2*n* = 32), *V. vinifera* (2*n* = 38), *C. acuminata* (2*n* = 21), *C. officinalis* (2*n* = 18), *C. controversa* (2*n* = 22), *C. wilsoniana* (2*n* = 20), *Solanum lycopersicum* (2*n* = 24), and *D. carota* (2*n* = 18) (Fig. 4A). To propose an updated ancestral monocot karyotype (AMK) structure, we first investigated the synteny between *C. officinalis* and the *n* = 7 AMK. The deconvoluted synteny plots distinctly delineated nine independent pairs of duplicated blocks spanning the entire *C. officinalis* genome, suggesting the preservation of nine ancestral regions corresponding to an *n* = 5 AMK (Fig. 4A). Specifically, these duplicated blocks accounted for 1246.5 Mb (57.7%) of the *C. officinalis* genome. Overall, most chromosomes within the *Cornales* species exhibit one-to-one syntenic relationships, with only localized intra-chromosomal inversions (around 5%), highlighting remarkable conservation of chromosomal architecture in these species. Furthermore, a pronounced 1:2 synteny ratio between the core eudicot karyotype (AEK) and *C. officinalis* reinforces the occurrence of an additional WGD event in *C. officinalis* following the WGT-γ event (Fig. 4B). From this ancestral state, the *C. officinalis* genome was subsequently remodeled through a lineage-specific WGD event (σ), resulting in an intermediate configuration of 2*n* = 18 chromosomes. This was then refined through a series of chromosomal rearrangements, specifically, seven fusions and six fissions, which culminated in the nine-chromosome modern karyotype observed today. These chromosomal changes resulted in a reduction of total genomic size from approximately 1520 Mb to the current 1200 Mb, emphasizing a substantial reduction in genome size following the lineage-specific event.

Given the extensive divergence observed within *Cornales*, reconstructing its karyotypic evolutionary history presents a formidable challenge. Nonetheless, by connecting the genome of *C. officinalis* to the AEK *via* syntenic gene blocks shared with the core eudicot karyotype (ACEK), and by employing mechanisms such as reciprocally translocated chromosome arms (RTAs), nested chromosome fusion (NCF), and end-end joining (EEJ), we can infer the origins and evolution of its chromosomes (Figs 4C and S11). The degree of genomic arrangement and chromosomal fusion in *Cornales* is significantly higher than that observed in the ACK lineage. Our analysis identified 23 chromosomal fusion events and 26 chromosomal fissions across four *Cornales* species (Fig. 4C), highlighting the dynamic and complex nature of their karyotypic evolution.

The AEK, originally with a base number of *n* = 7, underwent a WGD event (γ) that expanded it into 21 protochromosomes. Our results indicated that AEK chromosome fusion and fission events were most pronounced in *C. officinalis*, followed by *C. wilsoniana*, with *C. controversa* exhibiting the fewest alterations. Notably, chromosomes 2, 8, and 9 (Chr2, 8, and 9) in *C. officinalis* appear to have undergone specific fusion and fission events relative to other *Cornales* species. Chr2 appears to have originated *via* RTA derived from all AEK protochromosomes, with a total of 1 215 000 bp of duplicated sequences spanning across chromosomes 1 to 7. Chr3 arose through RTA events involving a greater number of protochromosomes than observed in other *Cornales* species, spanning 1 005 000 bp of duplicated sequences, representing 15.2% of the *C. officinalis* genome. Moreover, the formation of Chr9 shows distinct divergence patterns: in *C. wilsoniana*, it reflects the direct inheritance of ancestral chromosomes, accounting for 7.4% of the total genome size, whereas in *C. controversa*, the dominant mechanism is exon-to-exon joining (EEJ), with a segmental duplication region of 1 070 000 bp. This variation accounts for a 3.1% reduction in genome size in *C. officinalis* relative to *C. controversa*. Taken together, these results suggest that the evolutionary trajectory of fused chromosomes within *Cornales* is highly dynamic, contributing significantly to the variation in genome size observed among these species. In particular, chromosomes 2, 3, 8, and 9 of *C. officinalis* have undergone substantial centromeric and telomeric fusion events, with fusion regions spanning 920 000 bp in total, which have played a crucial role in accelerating chromosomal evolution within *Cornales* species.

### Recent bursts of *gypsy* retrotransposons contributed to the genome size expansion of *C. officinalis*

Within the *Cornales* genus, three closely related species, including *C. officinalis*, *C. wilsoniana,* and *C. controversa,* exhibit striking disparities in genome size. Notably, *C. officinali*s possesses a genome size of 2.96 Gb, which is nearly three times larger than those of the other two species. This substantial expansion is largely attributable to repetitive sequences, long recognized for their pivotal role in plant genome evolution [28]. In fact, repetitive elements constitute 75.04% of the *C. officinalis* genome, a proportion markedly higher than the 52.90% and 51.80% observed in *C. wilsoniana* and *C. controversa,* respectively (Table S7).

**Figure 5:**
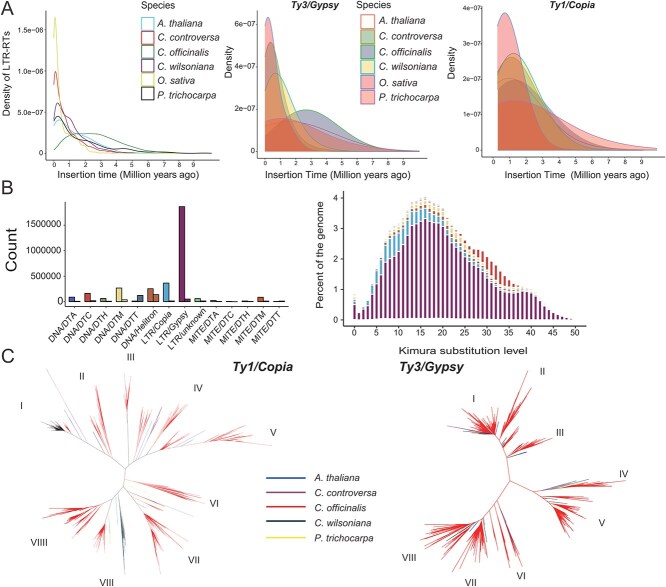
Distribution and evolution of transposable elements in the *Cornus officinalis* genome. **A** Distribution of insertion times for *Gypsy* and *Copia* retrotransposons across multiple plant species. **B** The percentage of different transposons and Kimura substitution level in *C. officinalis* was counted. Different TE subfamilies are classified and quantified, revealing their relative abundance in the genome. Phylogenetic relationships of different TE subfamilies in *C. officinalis* are shown, with all bootstrap values equal to 100, ensuring high-confidence branch support. **C** Phylogenetic relationships of *Gypsy* and *Copia* retrotransposon domains from five *Asterids* species. *A. thaliana* , *C. officinalis* , *P. trichocarpa* , *C. controversa* , *C. wilsoniana*.

A closer examination revealed that LTR sequences are the dominant repetitive elements in *C. officinalis,* with *Gypsy* elements representing 41.26% and *Copia* elements 9.66% of the genome. In comparison, *C. wilsoniana* displays lower levels of *Gypsy* (26.67%) and *Copia* (6.05%) elements, while *C. controversa* exhibits the lowest proportions (*Gypsy*: 21.87%; *Copia*: 6.61%). To investigate the temporal dynamics of LTR activity, we examined the *Ks* distributions across these species. The *Ks* values in *C. wilsoniana* and *C. controversa* reveal distinct peaks, indicative of relatively short bursts of transposon activity. In contrast, *C. officinalis* shows a broad and continuous *Ks* peak (Fig. 5A), suggesting a prolonged and sustained period of LTR insertion. Approximately 80% of the LTR insertions in *C. officinalis* occurred between 1 and 5 Mya, a pattern that stands in sharp contrast to the genomes of *C. wilsoniana* and *C. controversa*, where nearly 95% of LTR insertions are confined to the last 1 million years (Fig. 5A).

To further explore the evolutionary dynamics of LTRs in *C. officinalis*, we conducted a comprehensive phylogenetic analysis of these elements across *Cornales* species. Our results revealed that in *C. wilsoniana* and *C. controversa,* the *Gypsy* superfamily predominantly clusters into families I and IV (Fig. 5C). In contrast, the *Gypsy* elements in *C. officinalis* have undergone a relatively unique pattern of expansion, particularly within families II and VIII (Fig. 5C). However, this expansion trend was not observed for the *Copia* superfamily (Fig. 5C). The *Copia* elements in *C. officinalis* constitute only 13.2% of total LTR elements, compared to 23.4% in *C. wilsoniana* and 19.8% in *C. controversa*. Consequently, the continuous bursts of LTR-RT activity in *C. officinalis* are primarily driven by *Gypsy* amplification, which accounts for 56% of full-length LTRs and constitutes 29.65% of the genome assembly (Table S9). These findings underscore the pivotal role of *Gypsy* expansion in shaping the genomic architecture of *C. officinalis*.

The unequal recombination (UR) between LTRs results in the excision of the intervening sequence and the formation of solo LTRs [49]. To further investigate this phenomenon in *C. officinalis*, we estimated the relative rates of LTR- RT- associated UR within the genome. A total of 824 intact and 154 solo LTR elements were identified (Table S8). In plant genomes, UR is a common mechanism for eliminating LTR-RTs by creating solo LTRs. As expected, the ratio of intact to solo LTRs is notably higher in *C. wilsoniana* (2.3) and *C. controversa* (2.1) (Table S8). Altogether, these results indicate that a comparatively lower rate of UR-mediated LTR removal, in conjunction with a sustained burst of *Gypsy* RT families II and VIII, has substantially contributed to the genome expansion o bserved in *C. officinalis*.

### Genes with longer intron are highly expressed in *C. officinalis*

A significantly higher number of ultra-long introns were identified in the genome of *C. officinalis* compared to its *Cornales* counterparts (Fig. S12A). Specifically, 13 567 introns, making up 11.7% of the total introns, exceeded 12 kb in length-a feature rarely seen in *C. wilsoniana* and *C. controversa*. Moreover, the average intron length in *C. officinalis* was found to be 3.8 kb, significantly longer than the approximately 1.1 kb observed in the other two species (permutation test, *P* < 0.01; Fig. S12B). A clear positive correlation between the proportion of total intron/exon length and genome size was also observed (Fig. S12A and B; *r* = 0.65), suggesting that genome expansion in *C. officinalis* occurs not only in intergenic regions but also within genic regions.

To investigate the potential influence of introns on transcription, genes were stratified into distinct groups based on the length of their first introns (Fig. S12C and D). The analysis revealed that genes with longer first introns generally exhibited higher expression levels than those with shorter introns (Fig. S12D and E). Furthermore, a comprehensive assessment of various gene characteristics, including intron number and length, exon length, presence or absence of introns, presence of TEs, and total gene length, demonstrated a positive correlation between both intron number (*r* = 0.53) and intron length (*r* = 0.62) with gene expression levels (Fig. S12C and D). Collectively, these results shed light on the potential regulatory role of intron architecture in modulating the transcriptional dynamics and contributing to the genomic complexity of *C. officinalis*.

### Identification of key genes for the loganin biosynthesis pathway in *C. officinalis*

The biosynthesis of loganin in *C. officinalis* is of considerable interest due to its central role as a major source of iridoids. This biosynthetic process critically depends on the activity of LAMT, which utilizes loganic acid as its substrate. Through extensive homology searches and rigorous functional annotation, we identified 17 candidate genes encoding eight enzymes integral to the loganin biosynthesis pathway (Fig. S13A and Data S3). The transcriptome analysis results indicate that these genes are specifically expressed in fruits and leaves (Fig. S13B). Within this pathway, LAMT and SLS are particularly pivotal, as they play essential roles in the formation of monoterpene indole alkaloids (MIAs) [46, 50]. A higher median Ks for the LAMT and SLS genes suggests an ancient origin for these genes, which would otherwise have been actively evolving (Fig. S14). To further refine our identification of genes encoding LAMT- and SLS-like enzymes in *C. officinalis*, we constructed detailed phylogenetic trees incorporating all candidate genes from the *CYP72A* subfamily (Fig. S15), along with previously characterized *LAMT* and *SLS* sequences from *C. wilsoniana* and *C. controversa*.

Phylogenetic analyses showed that the *CYP750* and *CYP726A* gene families have undergone significant expansion in *C. officinalis* compared to *C. wilsoniana* and *C. controversa*. Notably, 61.48% of the *CYP726A* genes are concentrated on pseudochromosomes 3 and 8 (Fig. S13C–E), exhibiting a distinct nonuniform genomic distribution. Rather than being randomly dispersed, CYP450 genes in *C. officinalis* are organized into discrete biosynthetic gene clusters (BCGs) (Fig. S13D–E). Notably, nearly all of these clusters, except for clusters 2 and 6, consisted of members from no more than two *CYP726A* families (Data S4). Of particular interest is cluster 9, which is uniquely homogeneous, containing members from only a single *CYP726A* family. This distinct clustering pattern highlights a clear aggregation within the *CYP726A* subfamily, indicating a non-random spatial arrangement and family-specific organization (Data S4). Collectively, these findings suggest that the expansion and structural organization of the *CYP726A* subfamily have played a crucial role in directing the evolutionary trajectory of loganin biosynthesis in *C. officinalis*.

### Loganin biosynthetic genes are arranged in discrete biosynthetic gene clusters

BCGs are regions of a genome where genes involved in a biosynthetic pathway are located in close proximity [18]. To investigate the conserved gene sets involved in loganin biosynthesis, we categorized twelve plant species into two groups: loganin-producing plants (*C. controversa*, *D. involucrata*, *C. wilsoniana*, *C. officinalis*, *C. acuminata*, *C. roseus*, and *C. sinensis*) and nonloganin-producing plants (*V. vinifera, C. canephora*, *P. ginseng*, *D. carota*, and *S. lycopersicum*). Across these species, we identified a total of 374 metabolic BCGs. Notably, 64.8% of these BCGs were conserved across all species, while 128 BCGs were exclusive to the loganin-producing group (Data S5). In addition, several key synthetic genes, such as *LAMT*, *SLS,* and *BAHD,* were absent in the non-loganin-producing plants, emphasizing their potential roles in loganin biosynthesis. Focusing on the *C. officinalis* genome, our analysis revealed 37 potential secondary metabolite BCGs. These include nine saccharide clusters, twelve terpene clusters, one lignan cluster, one polyketide cluster, four alkaloid clusters, and ten predicted BCGs. Intriguingly, within a 10-kb region of contig C1693, three BADH family members were organized as a tandem gene array, suggesting that tandem gene duplication events have contributed to the assembly of genes associated with loganin biosynthesis. Moreover, by identifying BCGs that contained at least five orthologous families linked to loganin biosynthetic pathways, we classified 26 clusters as putative loganin-related BCGs (Data S5).

We identified a highly conserved BCG, designated C1693, in the *C. officinalis* genome, which encodes functionally characterized enzymes such as LAMT (*Co1.482* and *Co507.206*) and SLS (*Co1519.29*), in addition to cytochrome P450 and BAHD acyltransferase proteins (Fig. S16A). Syntenic analyses revealed that the loganin biosynthesis cluster is highly conserved across plant genomes (Data S6). Phylogenetic tree analysis further confirmed that approximately 3.2 Mya, a *Cornus*-specific gene duplication event led to the emergence of two distinct types of *LAMT* genes (Data S6). Moreover, genes within the C1693 cluster involved in loganin biosynthesis display pronounced tissue-specific expression patterns, with *Co07G1482*, *Co07G1487*, and *Co07G1241* being highly expressed in fruit (Fig. S16C). Further synteny analysis revealed a conserved syntenic region at the C1693 locus in both *C. wilsoniana* and *C. controversus,* which harbors several functionally characterized genes, including cytochrome P450s, terpene synthases, and BAHD acyltransferases (Fig. S16B). Together, these results suggest that the loganin BCGs may have been formed in *Cornus* through gene duplication events.

Although the *C. acuminata* genome shows substantial collinearity and sequence similarity with that of *C. officinalis*, it also maintains conserved BCGs collinearity at the C1693 locus (Fig. S16A). However, terpene synthase orthologus, typically conserved in loganin-producing plants, are absent in *C. acuminata*, implying a loss or modification of the terpene biosynthesis pathway in this species. Detailed analyses revealed that 17 amino acid residues (E91, A95, L99, S101, D107, D113, S116, P117, G118, G121–122, K124, H126, I129, Q131, N136, and Q316) in the *C. acuminata* orthologue exhibit alterations that likely compromise their functionality (Fig. S17A). The apparent absence of a fully functional LAMT in the *C. acuminata* genome likely impairs its ability to convert loganic acid into loganin, highlighting the evolutionary divergency in longanin biosynthesis pathways among these closely related species.

### Catalytic properties of CoLAMTs in loganin biosynthesis

**Figure 6:**
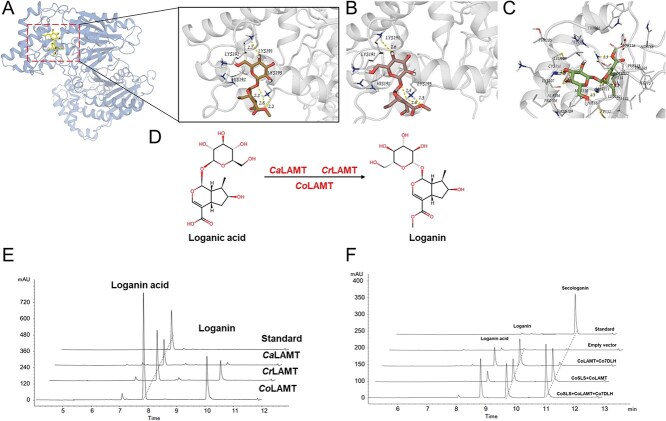
Structural and catalytic characterization of CoLAMT and its variants in loganic acid conversion to loganin. **A** Overall structure of CoLAMT, with the substrate loganic acid and S-adenosylmethionine (SAM) depicted in stick representation. The inset shows a detailed view of key catalytic residues (Lys191, His192, and His195) interacting with the loganic acid substrate at the active site, with hydrogen bond distances indicated (in Å). **B** Binding mode of loganic acid in the active site of CrLAMT, highlighting the distinct hydrogen bonding network and shorter interaction distances compared to CoLAMT, suggesting differences in substrate recognition and catalytic efficiency. **C** Active site interactions of loganin in the CaLAMT enzyme, showing key residues (Trp84, Phe113, His107) involved in substrate positioning and the orientation of the methyl group acceptor. Distances of hydrogen bonds are provided. **D** Proposed reaction pathway for the conversion of loganic acid to loganin by CaLAMT, CrLAMT, and CoLAMT, illustrating the methylation step catalyzed by these enzymes. **E** High-performance liquid chromatography (HPLC) profiles for the production of loganin by CaLAMT, CrLAMT, and CoLAMT using loganic acid as the substrate. The chromatograms show a marked increase in loganin production by CoLAMT, with peak intensities significantly higher than those of CaLAMT and CrLAMT, indicating superior catalytic efficiency of CoLAMT. **F** HPLC analysis of a coupled enzymatic reaction using CoSLS and CoLAMT. The co-incubation of CoSLS with loganic acid and CoLAMT results in efficient production of loganin and secologanin. In comparison, the reaction with CoSLS alone and loganin generates only secologanin, indicating that CoLAMT is essential for the full catalytic conversion.

Previous work in *C. acuminata* identified CaLAMT as a key methyltransferase responsible for the hydroxylation of loganic acid. To extend these findings, we identified three putative LAMT homologs, *Co1.482*, *Co507.206*, and *Co1486.99*-in *C. officinalis* via homology search and phylogenetic analysis. To investigate the mechanisms underlying the region-selective hydroxylation preference of *CoLAMT*, we constructed 3D structural models for these candidates using the AlphaFold3 protein structure prediction tool [51]. Comparative analysis of the catalytic pockets of CoLAMT, CaLAMT, and CrLAMT revealed notable differences attributed to variations in specific structural elements (His192, Lys191, and Lys195) (Fig. 6A-C). These differences primarily stem from distinct orientations of an α-helical region (residues 120–138), a B loop-ring structure (residues 141–176) (Fig. S16A), and the loganic acid binding region (residues 180–201). In molecular docking simulations, we found that the distance between the Lys195 residue of *CoLAMT* and the C-9 position is greater than that in *CaLAMT*, which may facilitate the deprotonation of the hydroxyl group, thereby initiating the methyltransferase reaction (Fig. 6A). This structural difference explains the substrate specificity of *CoLAMT* for the 9-OH site and aligns with its higher catalytic efficiency at this specific methylation site. In conclusion, these structural features, particularly the configuration of the catalytic pocket, confer distinct substrate specificity to *CoLAMT* and reveal the molecular mechanism of C-9 site selectivity in loganin biosynthesis (Fig. 6A–C). This structural arrangement accounts for the observed substrate preference of *CoLAMT* for the 9-OH site, which correlates with its higher catalytic efficiency in this specific methylation site (Figs S17B and S18). These findings highlight the structural features, particularly in the configuration of the catalytic pocket, which confer substrate specificity to *CoLAMT*, providing nuanced insights into the molecular mechanisms regulating loganin biosynthesis in *C. officinalis*.

To elucidate the functional roles of enzymes involved in loganin biosynthesis, we used a co-expression system to simultaneously express *CoLAMT*, *CrLAMT*, *CaLAMT,* and *CoSLS* in *Nicotiana benthamiana*. Initially, we constructed individual pBI121 vectors carrying the respective genes. Subsequent liquid chromatography-mass spectrophotometer (LC–MS) analysis confirmed that *CoLAMT* isoforms facilitated loganin production, as evidenced by the detection of an ion at m/z = 397 [M + H] (Fig. S19). High-performance liquid chromatography (HPLC) analysis demonstrated that only *CoLMAT* produced a novel product, indicating its unique catalytic role. As anticipated, *CoLAMT* exhibited robust enzymatic activity, efficiently converting loganic acid into loganin. Moreover, the transient co-expression of *CoLAMT* and *CoSLS* in *N. benthamiana* enabled the conversion of secloganin into strictosidine, thereby validating their cooperative function in the biosynthetic pathway. Collectively, these results highlight the distinct catalytic activities of *CoLAMT* and its key role in the conversion of loganic acid.

To gain a deeper understanding of the roles of *Co1.482*, *Co507.206*, and *Co1486.99* in the methylation of loganic acid to form loganin, we conducted detailed kinetic analyses of these enzymes. Under optimal conditions, *Co1.482*, *Co507.206*, and *Co1486.99* exhibited their maximum activities in 50 mmol/L Tris–HCl buffer (pH 8) at 45°C, with apparent *kcat/Km* values of 3381.49, 353.27, and 259.52 min/mol/L, respectively (Figs S20–S22; Table S9). Moreover, the loganin biosynthetic pathway was successfully reconstructed in *N. benthamiana*, achieving a loganin yield of 8.55 ± 0.11 μg/g dry weight (Table S10; Figs S19 and S24A–C). These results clearly demonstrate that although all three CoLAMTs are capable of catalyzing loganic acid methylation, *Co1.482* exhibits a catalytic efficiency approximately 8 to 16.5 times greater than its counterparts. This pronounced efficiency strongly suggests that *Co1.482* is the dominant enzyme mediating loganic acid hydroxylation *in vivo* (Figs. 6E-F). Overall, these results establish *Co1.482* as the principal hydroxylase in the loganin biosynthetic pathway and highlight its biotechnological potential for enhancing loganin production.

## Discussion

The evolution of specialized metabolite biosynthetic pathways is largely shaped by the emergence and diversification of enzymes that catalyze the pivotal steps in the synthesis of core metabolites. These enzymes drive the development of complex metabolic networks that enable plants to produce a wide range of bioactive compounds with essential ecological and medicinal properties [37]. In this context, we undertook an in-depth, integrative genomic study of *C. officinalis*, a species known for its rich array of specialized metabolites. By generating a high-quality, chromosome-scale genome assembly, we were able to construct a comprehensive resource that provides a clear view of the genomic architecture underlying its metabolic pathways. Utilizing this genomic framework, we focused on the loganin biosynthesis pathway in *C. officinalis*. Through the identification and characterization of the critical genes involved in loganin synthesis, our study sheds light on the molecular mechanisms that underlie the production of specialized metabolites in this species. This work opens new avenues for further exploration into the genetic and enzymatic determinants that enable *C. officinalis* to produce its diverse bioactive compounds, which have broad implications for plant biology, biotechnology, and medicinal research.

### Transposable element insertion events contribute to the expansion of *C. officinalis* genome

TEs are a major driver of genome expansion in plants [29, 52]. These mobile DNA sequences can move within a genome and often proliferate, leading to significant increases in genome size over evolutionary timescales [52, 53]. We observed that the *C. officinalis* genome is significantly larger than that of its closely related species, with extensive intergenic regions accounting for 75.4% of its total genome, 59.3% of which are enriched with TE repeats. Notably, the *Gypsy* elements in *C. officinalis* are particularly diverse, encompassing not only families II–VIII but also a distinct, species-specific family (family I). *Gypsy* elements are known to rapidly amplify *via* replicative mechanisms [54, 55], and their swift proliferation can induce genomic structural changes, including insertions, deletions, and rearrangements, which contribute to the diversification of genetic traits [46, 56]. Conversely, plant species typically counterbalance TE expansion through various elimination mechanisms such as RNA interference (RNAi), DNA methylation, and histone modification, all of which act to silence TEs and prevent their uncontrolled proliferation. Additionally, selective pressures may lead to the progressive degradation of TE sequences over time. However, the relatively low clearance efficiency in *C. officinalis* appears to be an important factor contributing to its genome expansion.

Despite the pronounced expansion of intergenic regions, introns are also notably elongated, contributing significantly to the overall increase in genome size in *C. officinalis*. Similar observations have been made in species like *Picea tabuliformis* and *P. asperata*, where super-long introns substantially contribute to their larger genomes [57, 58]. These elongated introns are enriched with repetitive sequences, particularly *Gypsy* retrotransposons, suggesting a potential role in driving genome evolution and structural variation. However, despite their considerable length, these introns do not appear to exert deleterious effects on plant growth and development [49, 59].

Our results revealed that genes with longer introns consistently exhibit higher expression levels compared to those with shorter introns (Fig. S12D and E). This pattern implies that longer introns may serve as reservoirs for regulatory elements, such as enhancers, silencers, and TF binding sites, which modulate gene expression and regulation. Notably, several highly expressed genes associated with stress resistance were identified, suggesting that intron elongation, while contributing to genome expansion, may also enhance evolutionary adaptability and facilitate regulatory diversification within the species.

### BGCs are essential for the biosynthesis of loganin

BGCs are essential modules in the biosynthesis of secondary metabolites, providing a framework for the coordinated production of bioactive compounds [60]. Our study identified 374 metabolic BGCs across 12 plant species, with 64.8% of these clusters being highly conserved across most species, indicating their fundamental role in specialized metabolism. Notably, 113 gene clusters are exclusively found in loganin-producing plants, suggesting that these clusters may play a specific role in loganin biosynthesis. It is worth mentioning that the genome of *C. officinalis* contains several metabolic gene clusters associated with secondary metabolism, including glycosides, terpenes, lignans, polyketides, and alkaloids, highlighting the complexity of its metabolic network.

We identified several key biosynthetic genes, such as *LAMT*, *SLS*, and *BAHD*, within the loganin-related gene clusters (Fig. S16A and B). Expression studies show that *CoLAMT* is highly expressed in fruit and leaf tissues, with levels closely correlating to loganin accumulation. Comparative genomic analysis further reveals significant regional conservation of these genes, particularly among loganin-producing plants. Consistent with the taxol-associated BGCs in the *Taxus* genome, the BCGs in *Cornus* are large and relatively diffuse, located within a broader 483 Mb biosynthetically rich region. The co-localization of multiple key genes involved in distinct steps of loganin biosynthesis within a confined genomic region suggests the presence of a potential coordinated regulatory mechanism governing their transcription. A promising direction for future research is to determine whether these genes constitute a larger-scale ‘supergene cluster’ that facilitates coordinated expression and enhances the overall efficiency of loganin biosynthesis.

Although the genome of *C. acuminata* exhibits high-gene collinearity and sequence similarity with that of *C. officinalis* in most regions, it lacks typical terpene synthase homologs in the C1693 region, suggesting that it may have lost or modified the terpene biosynthesis pathway involved in loganin biosynthesis. We found that mutations in key genes of *C. acuminata*, particularly in the *LAMT* gene, impair its ability to efficiently convert loganic acid into loganin. Therefore, the absence or functional loss of loganin biosynthesis in *C. acuminata* reveals an evolutionary divergence in biosynthetic pathways between these two species. In addition, we explored the potential application of C1693 in bioengineering.

Forty-eight hours after agroinfiltration, *N. benthamiana* leaves transiently expressing *CoLAMT* produced more than ten times the amount of loganin compared to those expressing *CaLAMT*. Specifically, the *CoLAMT*-expressing leaves produced 280 mg/L of loganin, whereas the *CaLAMT*- expressing leaves produced only 40 mg/L. Notably, the OD600 values of the two strains were similar 2.3 for *CoLAMT* and 2.2 for *CaLAMT-*indicating no significant difference in overall growth (Fig. S23). This result highlights the great potential of the C1693 region for future applications in bioengineering-based loganin production. Future research will focus on using genome-editing techniques, which may pave the way for the development of high-yielding crops or bioengineered systems for producing bioactive compounds.

### Efficient LAMT depends on its substrate specificity and catalytic efficiency

The catalytic efficiency of enzymes is dictated by both substrate specificity and intrinsic catalytic activity, which together govern the yield and metabolic flux within biosynthetic pathways [61, 62]. In plant secondary metabolism, a multitude of enzymes, including phenylalanine ammonia-lyase, glycosyltransferases, and P450 monooxygenases, function synergistically to synthesize complex molecules such as alkaloids, flavonoids, terpenoids, and phenolic compounds [61–65]. Even subtle mutations within these pivotal enzymes can profoundly influence their substrate affinity and catalytic efficiency [66], thereby modulating the accumulation of downstream metabolic products. For instance, mutations in *CaLAMT*, a key enzyme in loganin biosynthesis, have been demonstrated to alter its substrate specificity and catalytic efficiency, consequently affecting the production levels of this vital metabolite. Furthermore, the functional diversity of LAMT enzymes provides plants with an adaptive mechanism to balance energy allocation, optimizing the utilization of metabolic resources.

In *C. officinalis*, mutations within CoLAMT induce structural alterations in the ligand-binding pocket (Fig. S16B), which subsequently modulate its catalytic activity. Through transient expression of *CoLAMT* in *N. benthamiana* leaves, coupled with functional assays, it was confirmed that this enzyme proficiently catalyzes the conversion of loganic acid to loganin. The observed functional divergence within the ligand-binding region of CoLAMT likely accounts for its enhanced catalytic efficiency, thereby streamlining the loganin biosynthesis pathway in *C. officinalis*. Molecular dynamics simulations, kinetic assays, and reaction rate comparisons showed that CoLAMT has a preference for C9 hydroxylation (Fig. S24 and S25). Based on these simulations, we hypothesize that the absence of C9 hydroxylation leads to a larger positional shift of the hydroxyl group, resulting in reduced catalytic activity.

Notably, CoLAMT exhibits pronounced regioselectivity toward the C9′-OH group, achieving a conversion rate of 69.1%, which is substantially higher than the 43.1% observed for CaLAMT (Fig. S25; Table S11). This functional divergence enables *C. officinalis* to utilize fewer metabolic steps in loganin biosynthesis compared to *C. acuminata*. Molecular docking experiments and enzyme catalytic rate comparisons confirmed that CoLAMT has a preference for C-9 hydroxylation. Mutations in CoLAMT residues (Lys191, His192, and Lys195) resulted in a loss of catalytic activity towards the substrate loganin, indicating that these residues play an important role in determining C9-specific catalytic activity. Additionally, CoLAMT exhibits an anti-cancer activity that is an order of magnitude higher than that of CaLAMT against various cancer cell lines. The enhanced catalytic efficiency is likely attributable to increased enzyme stability and augmented substrate affinity, further bolstering CaLAMT’s performance within the loganin biosynthetic pathway.

This functional divergence markedly elevates the metabolic efficiency of *C. officinalis*, thereby promoting the biosynthesis of secondary metabolites. The functional characterization of CoLAMT fills a previously missing step in the loganin biosynthetic pathway, opening new possibilities for protein engineering of CoLAMT through enzymatic approaches both *in vivo* and *in vitro*. These mechanistic insights provide a foundation for the directed modification of LAMT, offering novel protein scaffolds for anticancer drug screening and future biotechnological applications.

## Materials and methods

### Plant materials

The study was conducted using a healthy female *C. officinalis* tree located in the Qinling Mountain National Nature Reserve, Foping City, Shaanxi Province, China (113° 89′ 55′′ N, 28° 26′ 32′′ E). In May 2021, young leaves were collected from this tree for DNA extraction and sequencing. To obtain high-quality samples for Bionano optical genome mapping, we utilized shoot-cutting technology to propagate seedlings from branches of the same tree. For Hi-C sequencing, genomic DNA was extracted from fresh leaves. RNA sequencing (RNA-seq) and methylation sequencing were performed on five different tissues and organs (young leaves, old leaves, roots, seeds, and fruits), all harvested from the same individual. To preserve sample integrity, freshly collected tissues were immediately flash-frozen in liquid nitrogen and subsequently stored at −80°C until further processing for DNA and RNA extraction, which is described in Methods S2.

### Evaluation of genome size and heterozygosity

To determine the genome size of *C. officinalis*, we obtained fresh leaf samples and conducted flow cytometry analysis using MoFlo™ XDP flow cytometry (Beckman Coulter Inc., Chaska, MN, USA). Nuclei were extracted from young leaves using a woody plant buffer, followed by filtration through a 300-μm nylon mesh filter to ensure sample purity. The nuclei were then stained with 50 μg/ml propidium iodide (PI) and incubated at room temperature for 10 min before fluorescence intensity was measured using the MoFlo™ XDP system.

To accurately estimate the genome size of *C. officinalis*, we employed the *Zea mays* (2.18 Gb) genome as an internal reference standard in the flow cytometric analysis. Genome size was calculated using the formula: *N = P-sample/P-target × C*, where *P-sample* represents the fluorescence intensity of the *C. officinalis* nuclei, *P-target* is the fluorescence intensity of the reference standard, and *C* denotes the known genome size of *Z. mays.* The detailed process is described in Methods S1.

To estimate the genome size and genomic features of *C. officinalis,* we employed the standard *K*-mer analysis approach. High-throughput sequencing data were processed using Jellyfish v2 to generate *K*-mer frequency distributions. The *K*-mer depth was determined by identifying the highest peak in the frequency distribution. Genome size was then estimated using the formula: *G* = *K-mer-number*/*K-mer-depth*, where *G* represents the estimated genome size, *K-mer-number* corresponds to the total number of identified *K*-mers, and *K-mer-depth* denotes the peak frequency of *K*-mer occurrence [67].

### Genome sequencing

High-quality genomic DNA was extracted from young leaves using the DNAsecure Plant Kit (Tiangen, Beijing, China) following the manufacturer’s protocol. The integrity and purity of the extracted DNA were assessed using a Qubit 4 fluorimeter (Thermo Fisher, Waltham, MA, USA) and agarose gel electrophoresis. For genome sequencing, a library with a 350-bp DNA insert size was prepared using the Truseq Nano DNA HT Sample Preparation Kit (Illumina, San Diego, CA, USA). The library was sequenced on the Illumina HiSeq platform (Illumina). These short reads were primarily used for genome-assisted assembly and error correction [68]. The detailed genome assembly process is described in Methods S3.

To obtain long-read sequencing data, a 20-kb DNA library was constructed and sequenced using Oxford Nanopore Technologies (ONT) on the PromethION platform (Oxford Nanopore Technologies, Oxford, UK) [69]. For genome scaffolding and structural variation analysis, long, high-quality DNA molecules were labeled with the restriction enzyme Nt.BspQI and processed using the Saphyr™ system [70] (Bionano Genomics, San Diego, CA, USA). The labelled DNA was loaded onto a Saphyr chip for scanning, and data processing was conducted using Bionano’s hybrid-scaffold software (https://bionanogenomics.com/support/software-downloads/). The detailed genome annotation process is described in Methods S4.

For Hi-C sequencing, genomic DNA was first crosslinked using formaldehyde to preserve chromatin interactions [71]. The crosslinked DNA was then lysed and digested overnight with *Dpn*II. The resulting DNA fragments were biotinylated and ligated to form chimeric circular molecules, which were subsequently purified, physically sheared, and enriched. Library preparation for sequencing involved DNA fragment end repair, adapter ligation, and PCR amplification following standard protocols [72]. Two Hi-C libraries were generated and sequenced on the Illumina HiSeq PE150 platform (Illumina), generating 150-bp paired-end reads. To ensure high-quality sequencing data, we performed rigorous filtering: reads containing more than 20% low-quality bases (Phred score ≤ 5) or more than 10% ambiguous bases (N bases) were removed [73, 74]. The multiomics analysis detailed process is described in Methods S5–S12.

### Metabolic biosynthetic cluster genes prediction

For BCG analysis, we employed PlantiSMASH software (https://plantismash.bioinformatics.nl/) to identify biosynthetic BCGs within the *C. officinalis* genome. Gene models were annotated by assigning four-part Enzyme Commission (EC) numbers and MetaCyc reaction identifiers based on protein sequence data. The classification of predicted catalytic functions was performed using E2P2 (https://plantcyc.org/about/documentation/e2p2-description) [75]. The assigned EC numbers were then converted into the corresponding MetaCyc (v22.5) reaction identifiers for pathway inference and pathway database construction using PathoLogic software (v22.5) [76]. To refine and validate pathway predictions, we manually curated the database using SAVI software (https://savi.sourceforge.io/), filtering out false positives, redundant pathways, non-plant pathway variants, and pathways already incorporated within broader biosynthetic networks. The curated pathway database, along with assigned metabolic reactions and enzyme annotations from *C. acuminata* and *C. roseus*. Was used as input for the PlantClusterFinder software (https://github.com/carnegie/PlantClusterFinder). The *C. officinalis* genome annotation structure was integrated into the PlantClusterFinder pipeline following the developer’s recommendations [56].

To identify BCGs associated with MIA biosynthesis, the *C. officinalis* genome was mapped against the MIA protein database. A BCG was considered an MIA biosynthetic cluster if it contained one or more high-confidence genes involved in MIA biosynthesis. For comparative analysis, BCG synteny mapping was conducted to examine the conservation of *C. officinalis* BCGs with those of two other MIA-producing plants, i.e. *C. acuminata* and *C. roseus*. The statistical significance of conserved gene order was assessed using a one-sided Fisher’s exact test [77].

### Heterologous expression of *CoLAMT* in *N. benthamiana* leaves

For transient expression in *N. benthamiana*, the coding regions of *CoLAMT* and two recombinant genes were cloned downstream of the CaMv 35S promoter in the binary vector pEASY-T1. Each resulting construct was introduced into *Agrobacterium tumefaciens* strain GV3101 *via* electroporation. The transformed *A. tumefaciens* cells were grown at 28°C in LB medium supplemented with kanamycin (50 mg/L) and rifampicin (50 mg/L) until the culture reached an optical density of OD600 = 0.6. For transient expression, bacterial cultures were harvested and resuspended in 10 mM MES buffer containing 10 mM MgCl_2_ and 0.1 mM acetosyringone, adjusting to the final OD600 to 1.0. Agroinfiltration was performed by infiltrating bacterial suspension into the young leaves of 5-week-old *N. benthamiana* plants using a needleless syringe. Seven days postinfiltration, leaves were harvested for further analysis of *CoLAMT* expression.

### LAMT enzyme activity assay

The enzyme activity assay was performed by adding 2 μg of purified protein to 1 ml of enzyme reaction mixture, which contained 50 mM Tris–HCl (pH 8.0), 0.5 mM acetyl-CoA, 1 mM benzyl alcohol, and 0.01 mM MgCl_2_. The reaction mixture was incubated in a water bath at 30°C for 15 min. The reaction was terminated by adding 400 μl of methanol, followed by centrifugation at 20 000× g for 10 min. The resulting supernatant was collected for further analysis. Sample separation was performed using a Newmate™ C18 column (4.6 × 250 mm, 5 μm; Yuexu Ytecnology, Shanghai, China) at a column temperature of 35°C. The mobile phase consisted of acetonitrile (A) and water (B). The gradient elution program was set as follows: 0 min, 20% A; 3 min, 45% A; 4 min, 45% A; 8.5 min, 100% A; 12.5 min, 100% A. The flow rate was maintained at 1.2 ml/min, and the injection volume was 10.0 μl. The sampler temperature was set at 35°C, and detection was performed at 236 nm. The MS conditions used for UPLC-MS followed the same settings. The ion source spray voltage was set at 3.0 kV, and the capillary temperature was maintained at 300°C. The scanning range was set from m/z 150 to 1500. For the multiple reaction monitoring (MRM) mode, optimization of the declustering potential (DP) and collision energy (CE) values was performed. In the MS2 model, the daughter ion of loganin (m/z 389.38) was scanned. Protein concentration was determined using the A280 (nm) ultraviolet absorption method. The relative enzyme activity of CoLAMT was calculated by comparing it to CaLAMT as a reference.

### Molecular docking and structure analyze

To predict and analyze the tertiary structure of the LAMT protein, we utilized the Swiss-model (https://swissmodel.expasy.org/), an automated homology modeling server. For protein-ligand docking and binding energy calculations, we used AutoDock v 4.2.6. Docking simulations were conducted using the local search algorithm, with default parameters applied for energy minimization and conformational sampling. The binding affinities between LAMT and its respective substrates were estimated based on calculated docking scores and interaction energies. The detailed process is described in Methods S13.

## Supplementary Material

Web_Material_uhaf259
