# Supplementary material for: Chromosome-level genome assembly of Cornus officinalis reveals the evolution of loganin biosynthesis
Source: Hortic Res. 2025 Sep 24;13(1):uhaf259. doi: 10.1093/hr/uhaf259 (PMC12860560; doi:10.1093/hr/uhaf259)
Supplement: Web_Material_uhaf259 [file web_material_uhaf259.zip › Supplementary Figures 1-25.docx]

**Supplementary Figures**

**Figure S1.** Genome size and heterozygosity of *C. officinalis* estimated.

**Figure S2.** Venn diagram for orthologous protein-coding gene clusters in *C. sinensis*, *D. carota*, *C. acuminata*, *C. roseus*, and *C. officinalis*.

**Figure S3.** Syntenic relationship of *C. officinalis*, *V. vinifera* and *C. acuminata*.

**Figure S4.** Syntenic dot plots between *C. officinalis*, *V. vinifera* and *C. acuminata*.

**Figure S5.** NACs gene family evolution and duplication.

**Figure S6.** Expression patterns of *CoNAC* genes in response to stress treatments in various tissues of *Cornus officinalis*.

**Figure S7.** Morphology and physiology characteristics of WT, *84K NAC12-OE2*, and *84K NAC12-OE3* plants under drought stress.

**Figure S8.** KEGG pathway analysis of expanded gene families of *Cornus officinalis*.

**Figure S9.** Genome size and heterozygosity of *Cornus officinalis* estimated.

**Figure S10.** Differentially methylated regions between roots and fruits.

**Figure S11.** Ideograph of chromosome origin in *Cornus officinalis*.

**Figure S12.** Correspondence of expression levels and difference genomic regions.

**Figure S13.** Genes involved in loganin biosynthesis pathway.

**Figure S14.** Median synonymous substitutions per synonymous sites (*Ks*) distribution for genes associated with loganin biosynthesis pathways across MIA plants.

**Figure S15.** Phylogenetic tree of the candidate *LAMTs* and *SLSs* encoding genes identified in *Cornus officinalis* and seven MIA plants.

**Figure S16.** The evolution of monoterpene loganin biosynthesis in plants.

**Figure S17.** Alignment of amino acid sequence of *LAMT* encoding gene copies in *Cornus officinalis* and other species.

**Figure S18.** Alignment of amino acid sequence of SLS encoding gene copies in *Cornus officinalis* and other species.

**Figure S19.** EIC for 9-loganin in *Nicotiana benthamiana* leaves expressing Co1.482, Co507.206, and Co1486.99, with the infiltration of CoLAMT.

**Figure S20.** Biochemical properties of Co 1.482.

**Figure S22.** Biochemical properties of Co 507.206.

**Figure S22.** Biochemical properties of Co1486.99.

**Figure S23.** Comparison of the two types of LAMT on the production of loganin.

**Figure S24.** Identiﬁcation of *LAMT* biosynthetic genes in *Cornus officinalis.*

**Figure S25.** The surface presentation of CoLAMT and CaLAMT.


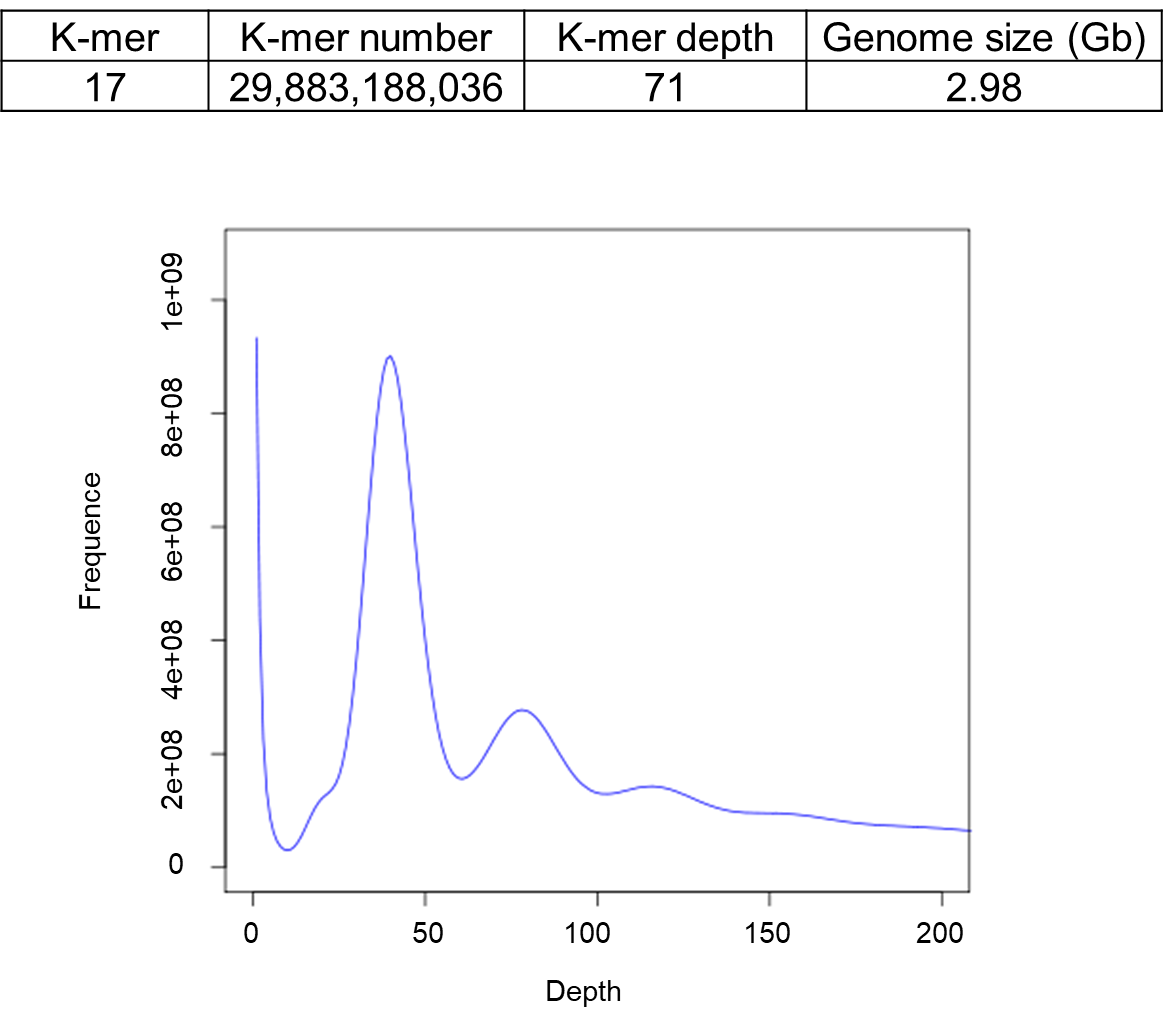
**Figure. S1. Genome size and heterozygosity of *C. officinalis* estimated.** The Figure. shows the frequency of 17 *K*-mers, we identified the peak of *K*-mers depth is 71. Genome size can be estimated as (total *K*-mers number) / (the volume peak). The genome size of *C. officinalis* was thus estimated as 2.98 Gb.


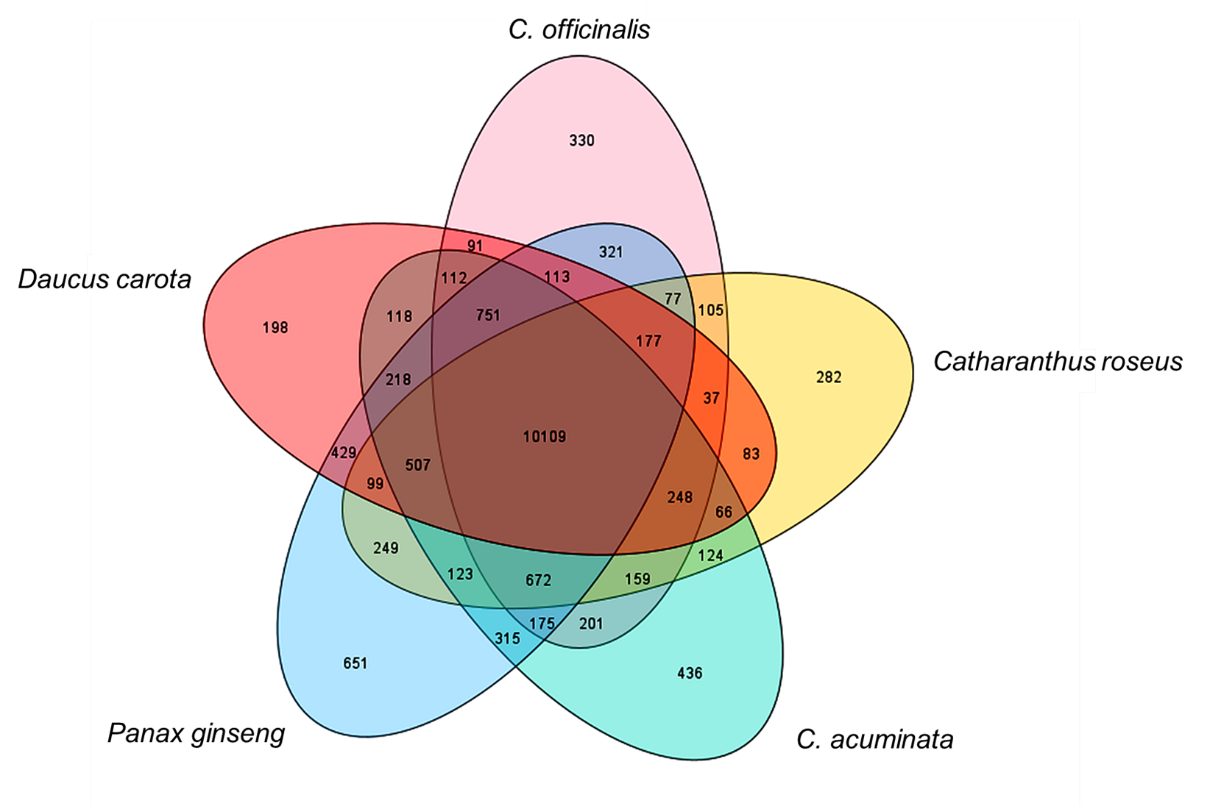
**Figure. S2.** **Venn diagram for orthologous protein-coding gene clusters in *C. sinensis*, *D. carota*, *C. acuminata*, *C. roseus*, and *C. officinalis*.**


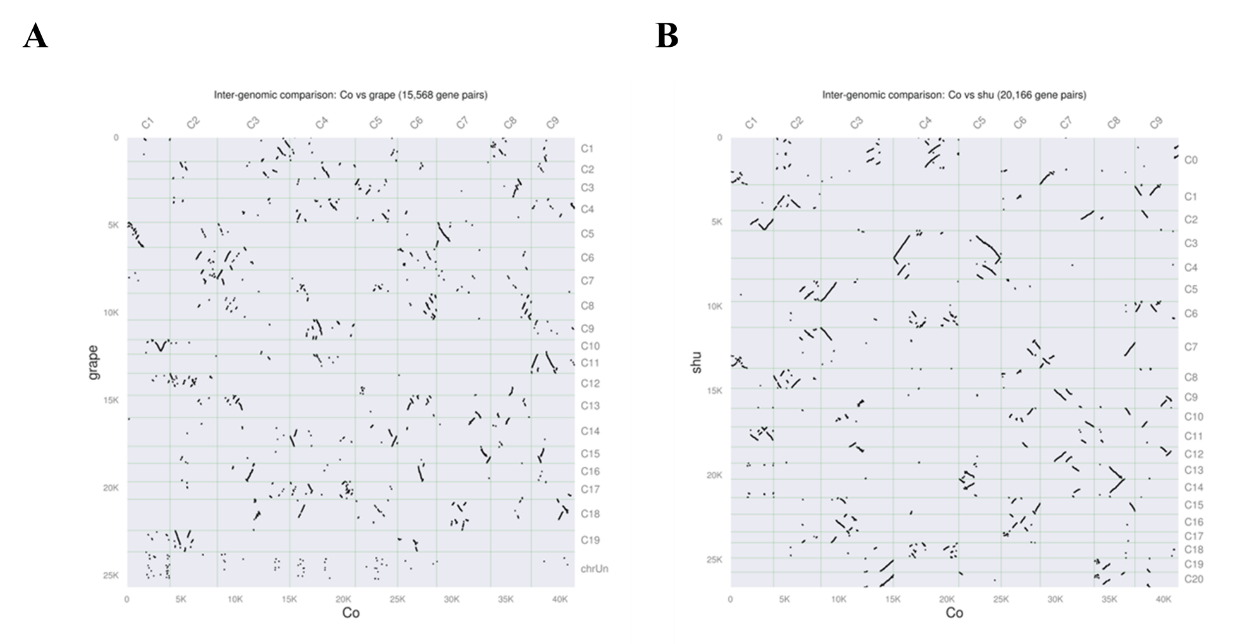
**Figure. S3. Syntenic relationship of *C. officinalis*, *V. vinifera* and *C. acuminata*.** **A.** Syntenic depth showed 2:1 relationship between *C. officinalis* and *V. vinifera* . **B.** Syntenic depth showed 1:1 relationship between *C. officinalis* and *C. acuminata*.


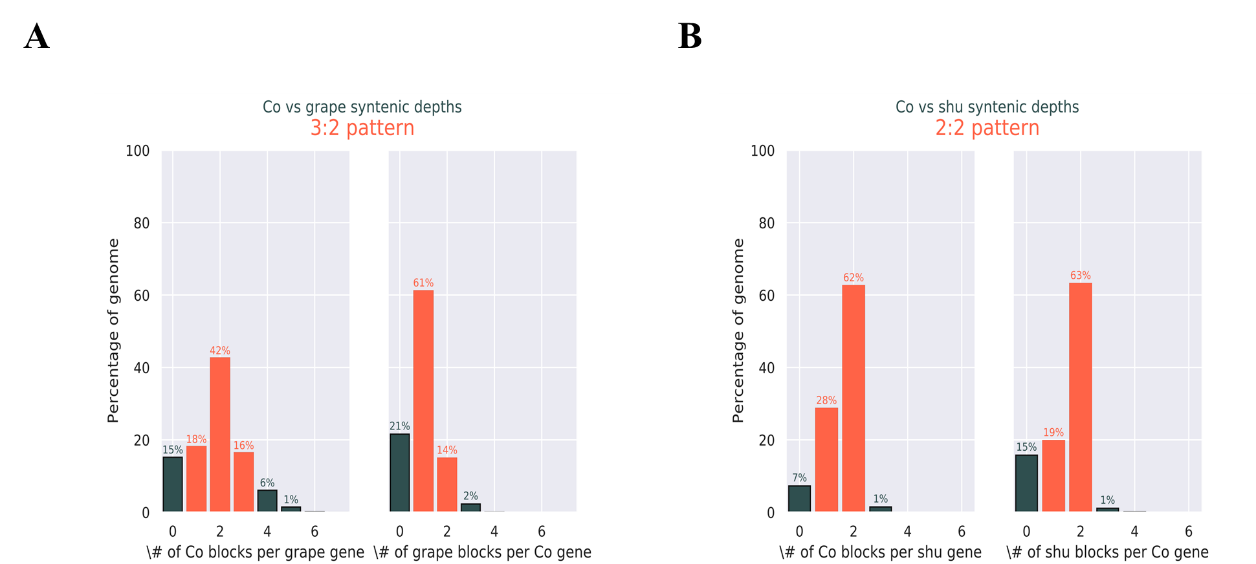
**Figure. S4. Syntenic dot plots between *C. officinalis*, *V. vinifera* and *C. acuminata*.** A. Intergenomic dot plot between *C. officinalis* and *V. vinifera*. B. Intergenomic dot plot between *C. officinalis* and *C. acuminata* .


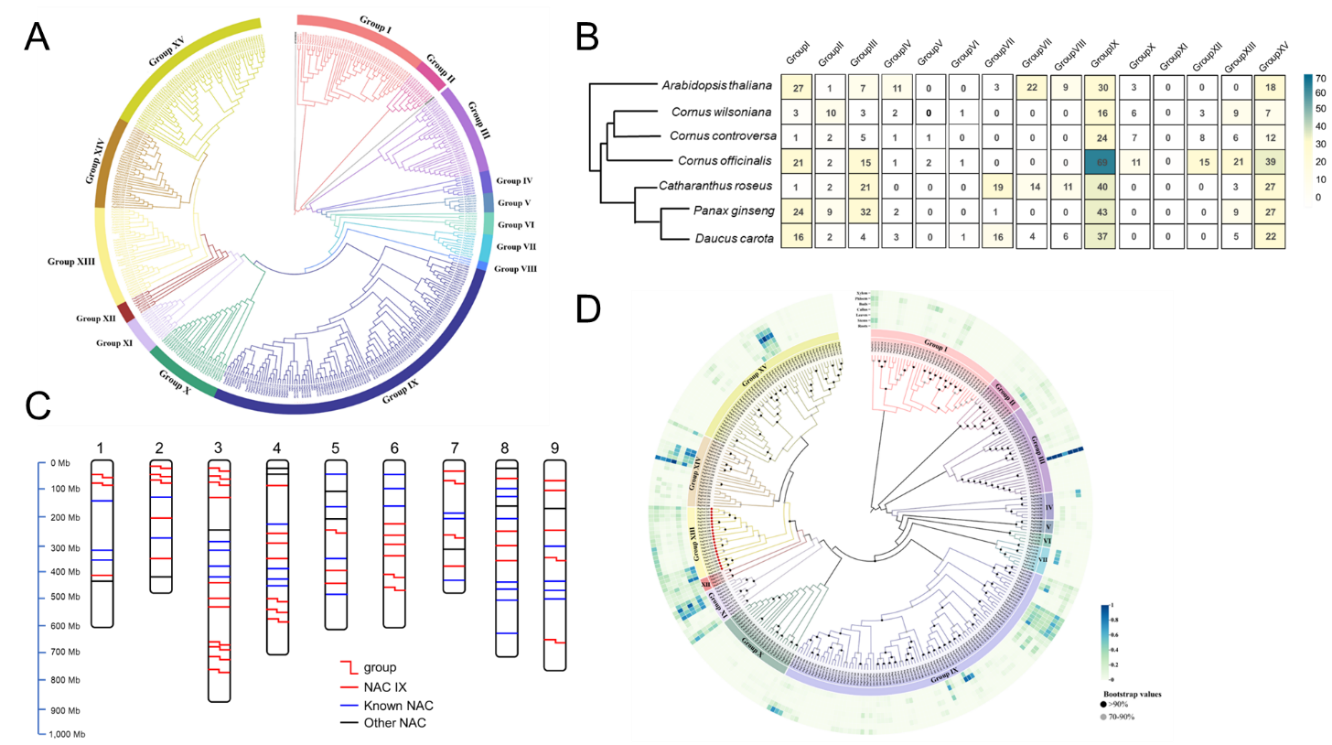
**Figure. S5. NACs gene family evolution and duplication. A.** Phylogenetic tree of the NACs genes identified in *C. officinali*s genome. Different colors indicate different NAC family members in *A. thaliana*, *C. acuminata* and *C. officinali*s genome. All branch bootstrap values are 100. **B.** The number of NAC gene family from different species. The color of blocks represent the NAC gene numbers. **C.** Chromosome localization of NAC genes of *C. officinali*s. The genes in red indicate members of NAC IX clade, which show several tandem repeat clusters. **D.** Expression profiles of the NAC transcripts on five tissues (fruit, leaf, steam, seed and root) of *C. officinalis*.


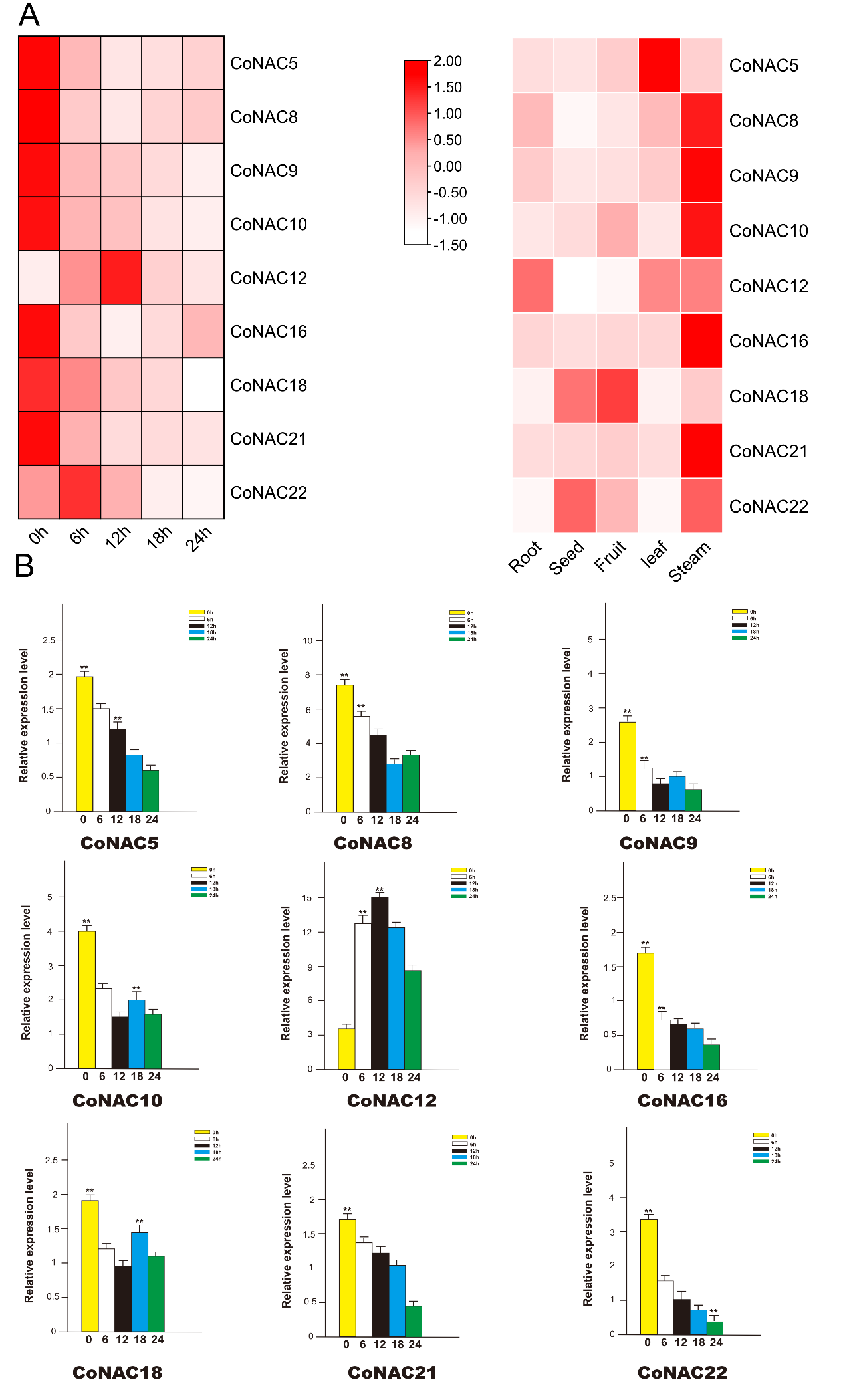


**Figure. S6. Expression patterns of CoNAC genes in response to stress treatments in various tissues of *C. officinalis*.** A. Heatmap showing the relative expression levels of CoNAC genes in different tissues (Root, Seed, Fruit, Leaf, Stem) of *C. officinalis* at various time points (0, 6, 12, 18, 24 hours) following treatment. The color scale represents the relative expression levels (log2 fold change) of 10 CoNAC genes, where darker red indicates higher expression levels. B. Quantitative RT-PCR analysis of CoNAC gene expression in different tissues (*C. officinalis*) at multiple time points post-treatment. The relative expression levels of CoNAC genes (CoNAC5, CoNAC8, CoNAC9, CoNAC10, CoNAC12, CoNAC16, CoNAC18, CoNAC21, CoNAC22) were measured using the 2^-∆∆Ct method. Data are presented as means ± SD (n = 3 biological replicates). Statistical significance was determined by one-way ANOVA followed by Tukey’s test (P < 0.01).


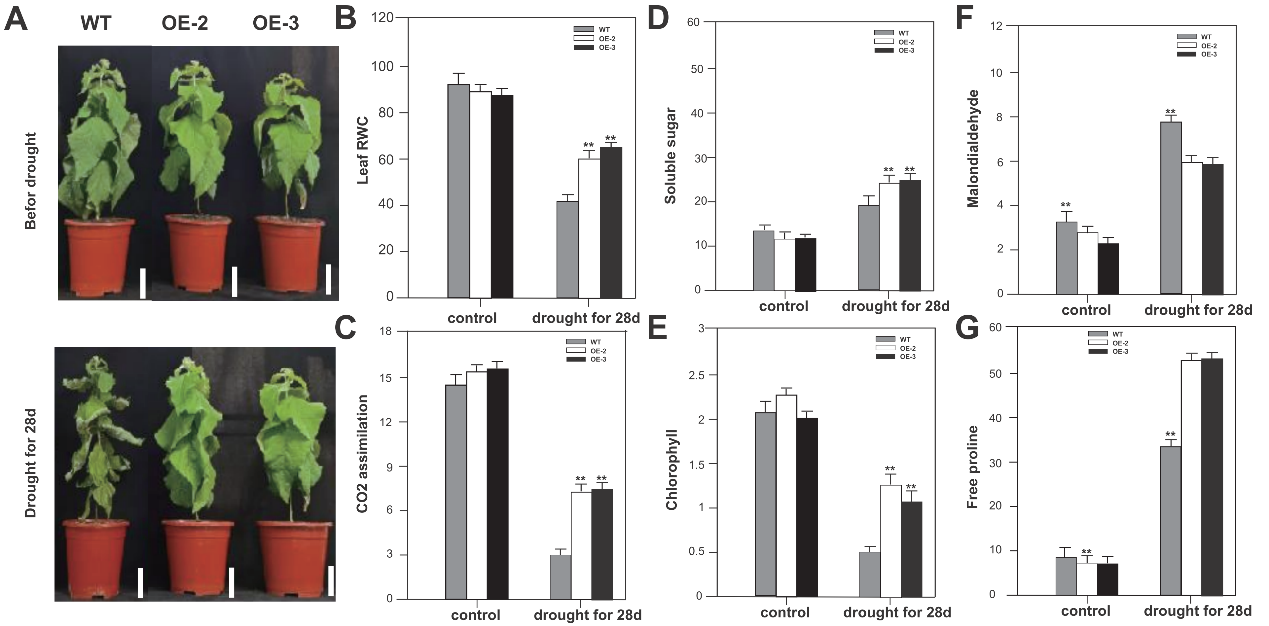


**Figure. S7. Morphology and physiology characteristics of WT, *84K* NAC12-OE2, and 84K NAC12-OE3 plants under drought stress.** A. The 3-month-old tissue cultured plants were planted in soil for 28 days (well-watered) and subsequently dehydrated until the soil water content reached 16 %. B-C. The leaf RWC and CO2 assimilation of WT, 84K NAC12-OE, and 84K NAC12-OE3 poplar lines under drought stress. Significant differences compared to the WT under mock or drought conditions were determined using Student’s t-test. Values represent the mean SD of three biological replicates (*, P < 0.05 and **, P < 0.01; n = 3). D. Soluble sugar content determined using the anthrone-sulfuric acid method. E. Total chlorophyll content extracted with 80% acetone and measured spectrophotometrically. F. Malondialdehyde (MDA) content, an indicator of lipid peroxidation, quantified by the thiobarbituric acid (TBA) method. G. Free proline content determined using the acid-ninhydrin method. All data represent mean ± standard deviation (SD) from three independent biological replicates (n = 3). Statistical analysis was performed using Student’s t-test. Asterisks indicate significant differences compared with WT under drought stress conditions (P < 0.01).


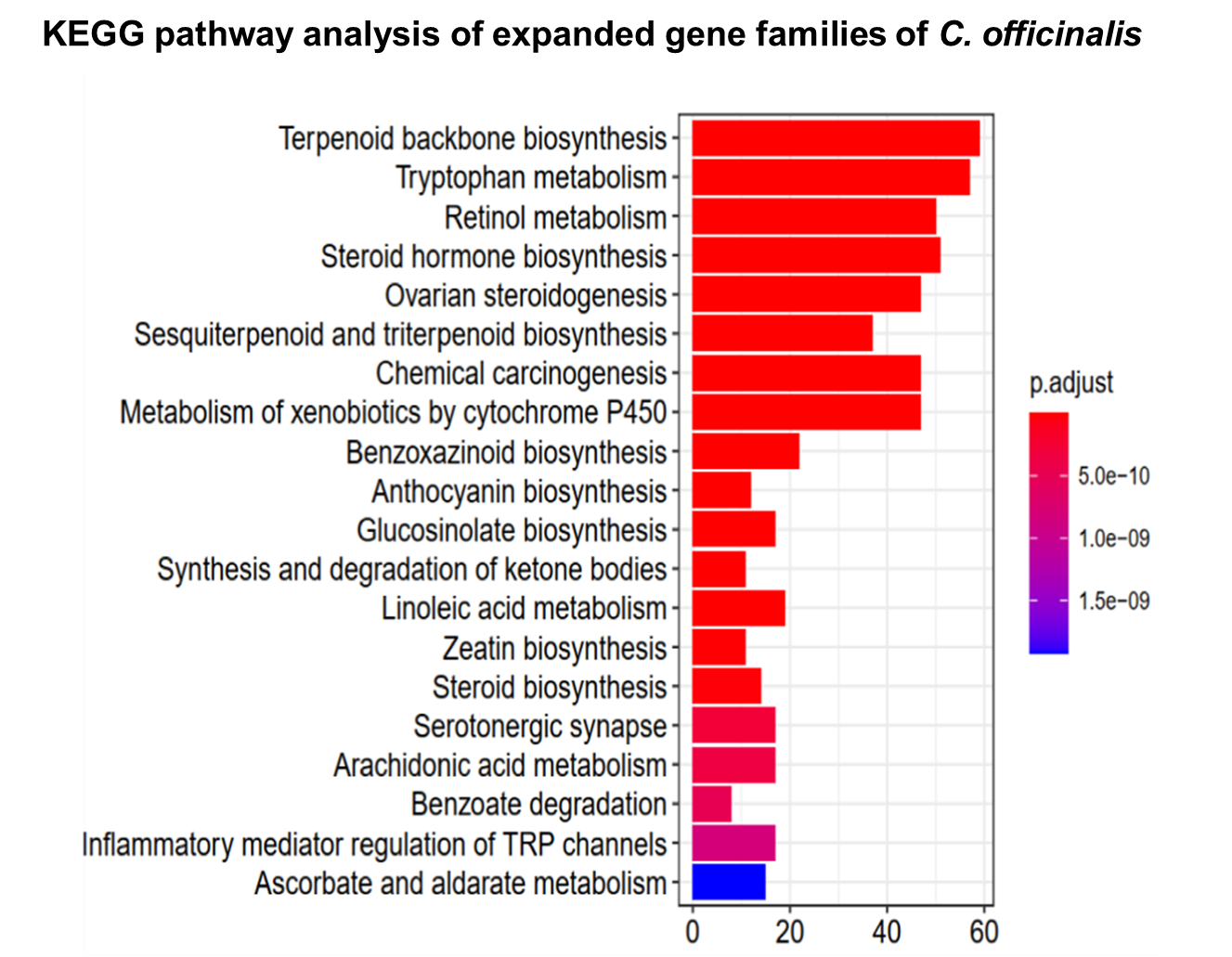
**Figure. S8. KEGG pathway analysis of expanded gene families of *C. officinalis*.** Color indicates the adjust *P* values for hypergeometric test and FDR adjustments.


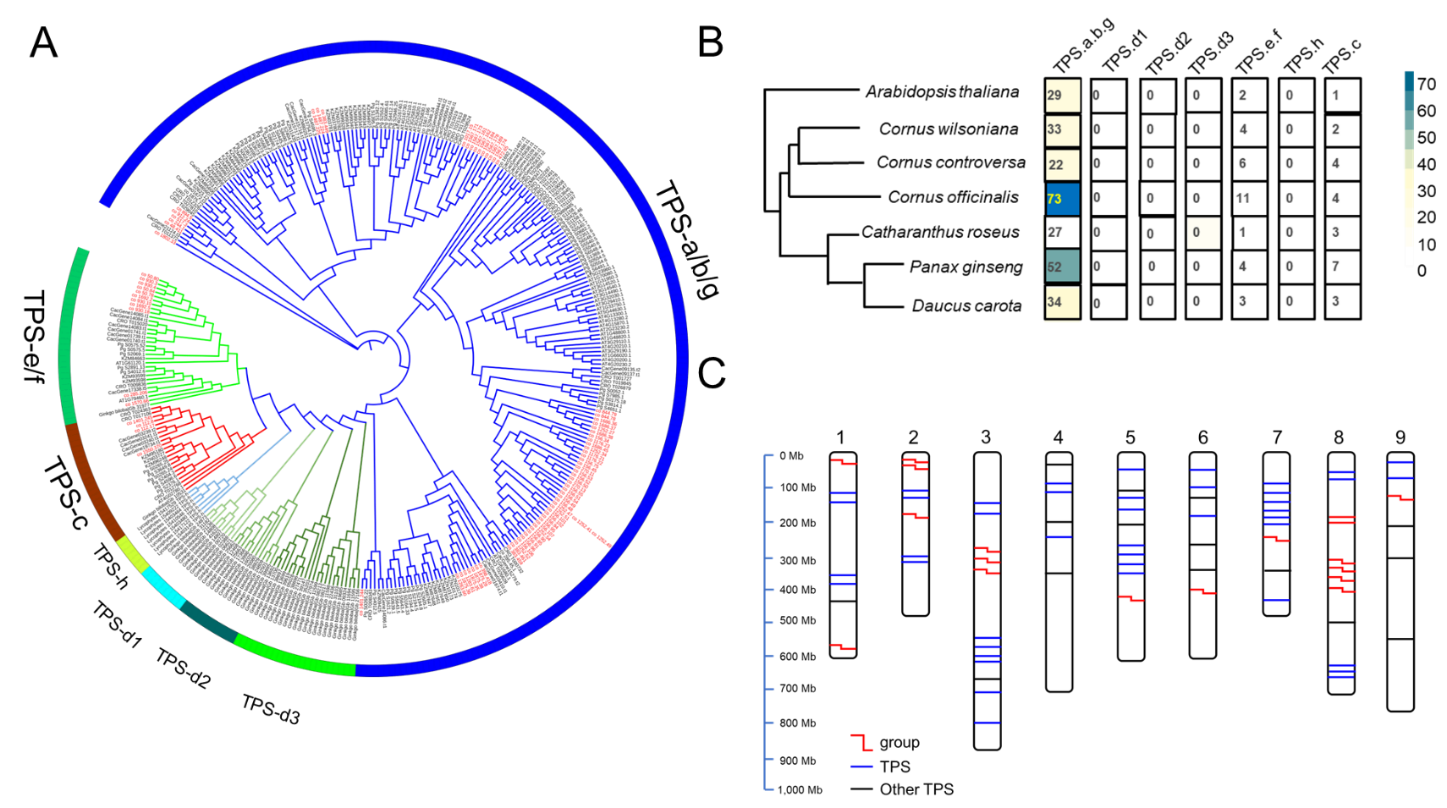
**Figure. S9. Genome size and heterozygosity of *C. officinalis* estimated.** A. Phylogenetic tree of the TPSs genes identified in *C. officinalis* genome. Different colors indicate different TPS family members in *A. thaliana*, *C. acuminata* and *C. officinalis* genome. All branch bootstrap values are 100. B. The number of TPS gene family from different species. The color of blocks represent the TPS gene numbers. C. Chromosome localization of TPS genes of *C. officinalis*. The genes in red indicate members of TPS IX clade, which show several tandem repeat clusters.


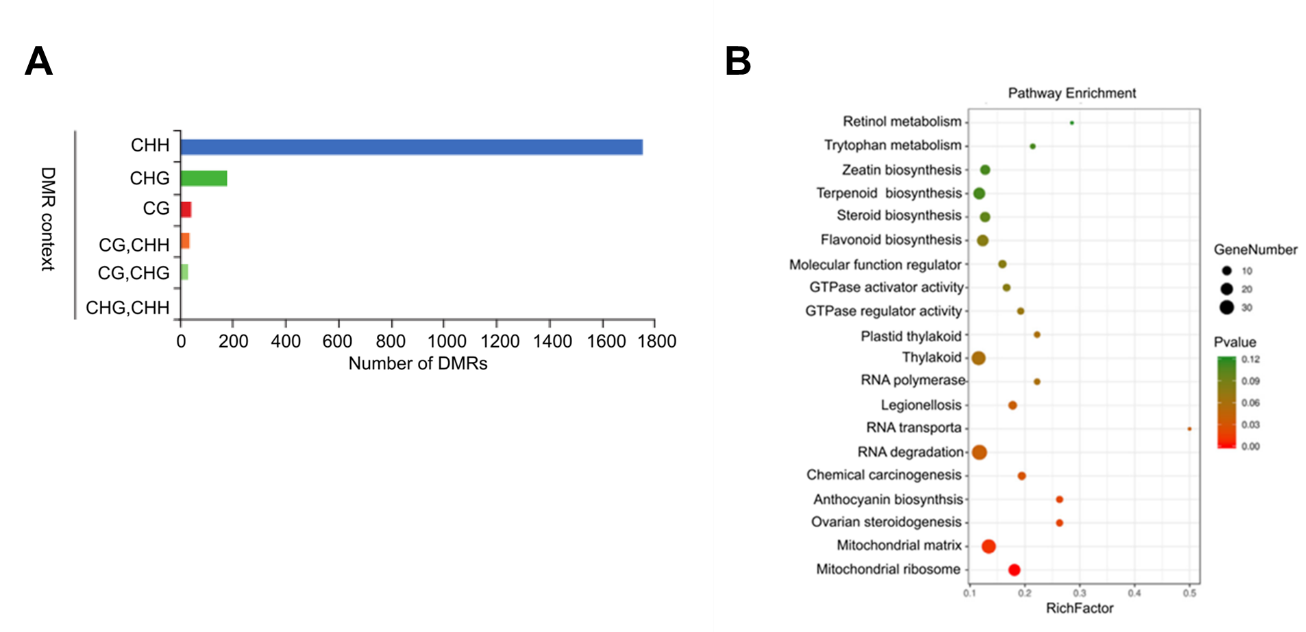
**Figure. S10. Differentially methylated regions between roots and fruits. A.** DMR content in samples of roots and fruits (CHH, n = 1794 DMRs; CHG, n = 188 DMRs; CG, n = 64 DMRs; CG and CHH, n = 56 DMRs; CG and CHG, n = 46 DMRs; CHG and CHH, n = 3 DMRs). Most of the DMRs (80.9%) were identified in the CHH context. **B.** KEGG enrichment analysis of DMRs in fruits.


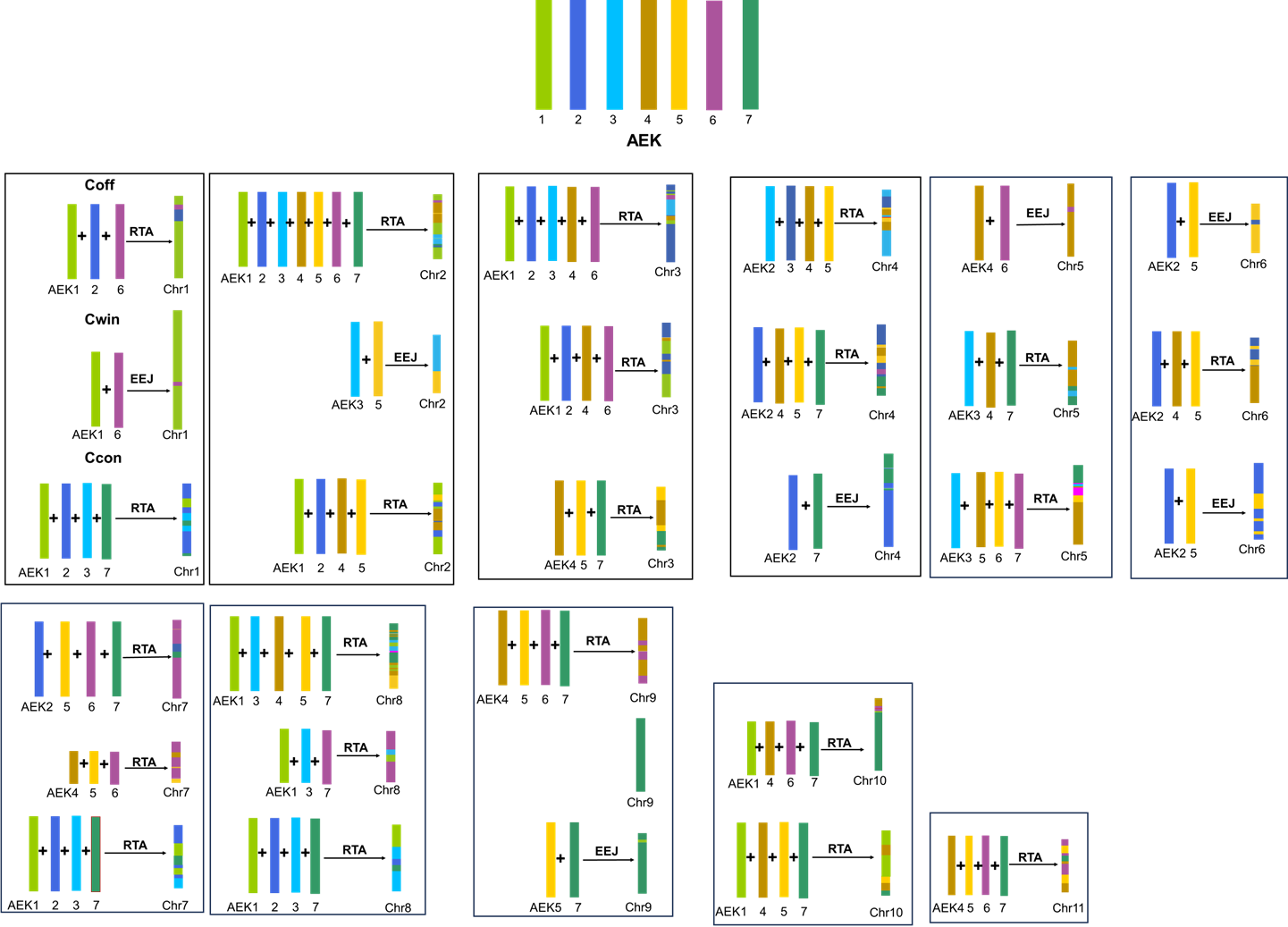
**Figure. S11. Ideograph of chromosome origin in *C. officinalis*.** The evolutionary history of each chromosome was inferred using WGDI. RTA, EEJ and NCF are the abbreviations for reciprocally translocated chromosome arms, end-end joining, and nested chromosome fusion. The line indicates chromosome fission, and the exchange icon indicates chromosome inversion. The colors represent different ancestral chromosomes. The protochromosome number of most recent common ancestor and chromosome number of extant sampled species are indicated using Roman numbers from up to down along.


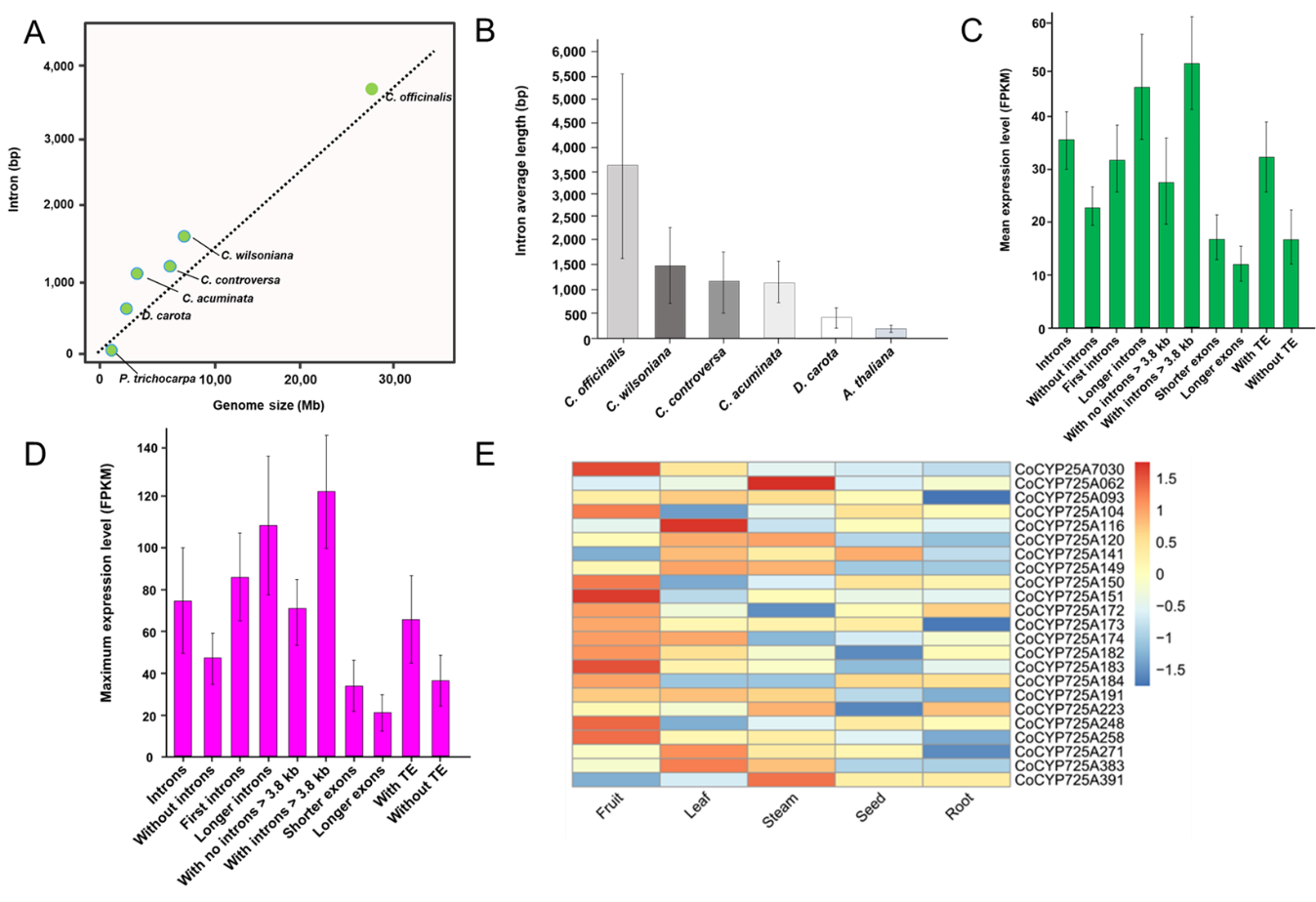
**Figure. S12. Correspondence of expression levels and difference genomic regions. A.** Dotplot shows correlation between intron length and genome size. **B.** The comparison of intron average length between *C. wilsoniana*, *C. officinalis*, *C. acuminata*, *D. carota*, *A. thaliana* and *C. controvers*. The color of blocks represents the different species. Error bars display the standard error. (C and D) Transcriptome-level distribution across different genomic regions. We divided the gene region into different regions based on the length of the intron, such as the first intron, long intron and whether there is a TE insertion region. The expression level of each gene was represented by the average expression level calculated based on transcriptomes. Error bars display the standard error. Expression distribution of different genomic regions, including first introns, other intron regions, and intron-less exonic regions. Expression levels were calculated from strand-specific RNA-Seq data using TPM normalization. Intron and exon annotations were derived from the curated reference genome, and read counts were assigned using featureCounts. Genes with high intron retention or ambiguous splicing were excluded from the analysis. **E.** Expression profiles for the intron resistant genes across five tissues (fruit, leaf, steam, seed and root) of *C. officinalis.*


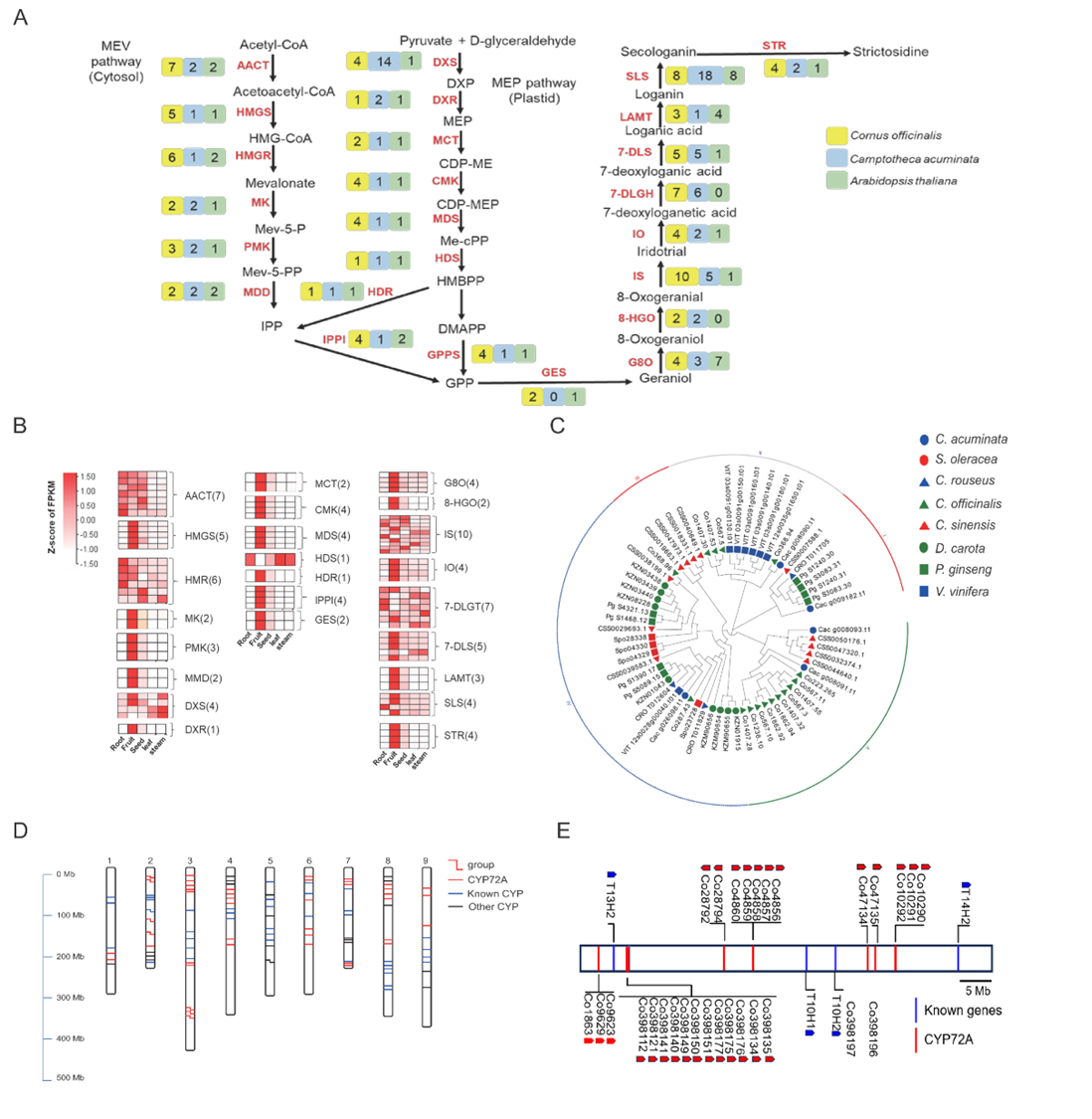
**Figure. S13. Genes involved in loganin biosynthesis pathway. A.** A simplified representation of the loganin biosynthetic pathway. GPPS, geranyl pyrophosphate synthase; GES, plastid geraniol synthase; G8O, geraniol 8-oxidase; 8-HGO, 8-hydroxygeraniol oxidoreductase; IS, iridoid synthase; IO, iridoid oxidase; 7-DLGT, UDP-glucoseirid oidgluco syltransferase; 7DLH, 7-deoxyloganicacid hydroxylase; LAMT, loganic acid methyltransferase; SLS, secologanin synthase; STR, strictosidine synthase. **B.** The expression profiles of the genes involved in terpene biosynthesis pathways in the of *C. officinali*s. Top hits for pathway genes identified by BLAST search and pathway genes in the co-expression network. The expression value for each gene is indicated in color on a log10 (FPKM + 1) scale for five tissues: root, fruit, seed, leaf, stem. **C.** Phylogenetic tree of CYP72 gene family for *C. officinalis* and seven other plants. Red circle represents *S. oleracea*; blue circle represents *C. acuminata*; green circle represents *D. carota*; red triangle represents *C. sinensis*; blue triangle represents *C. rouse*; green triangle represents *C. officinalis*; blue square represents *V. vinifera*; green square represents *P. ginseng*. **D.** Distribution of CYP450 genes on the nine pseudochromosomes. Each short line on the pseudochromosomes represents a CYP450 gene. CYP72As, and the other CYP450s are marked by red and blue lines, respectively. The known CYP450s in the loganin biosynthesis pathway (known CYP) are shown in blue. The CYP450 groups (≥ 7 CYP450 genes and ≤ 5.26 Mb of gene spacing between two adjacent CYP450s) are labelled outside of the corresponding positions on the pseudochromosomes. **E.** Map of CYP72As located in pseudochromosome nine. CYP72As and the other genes are marked by red and blue lines, respectively. The known CYP450s in the paclitaxel biosynthesis pathway (known CYP) are shown in blue. The arrows show gene orientations.


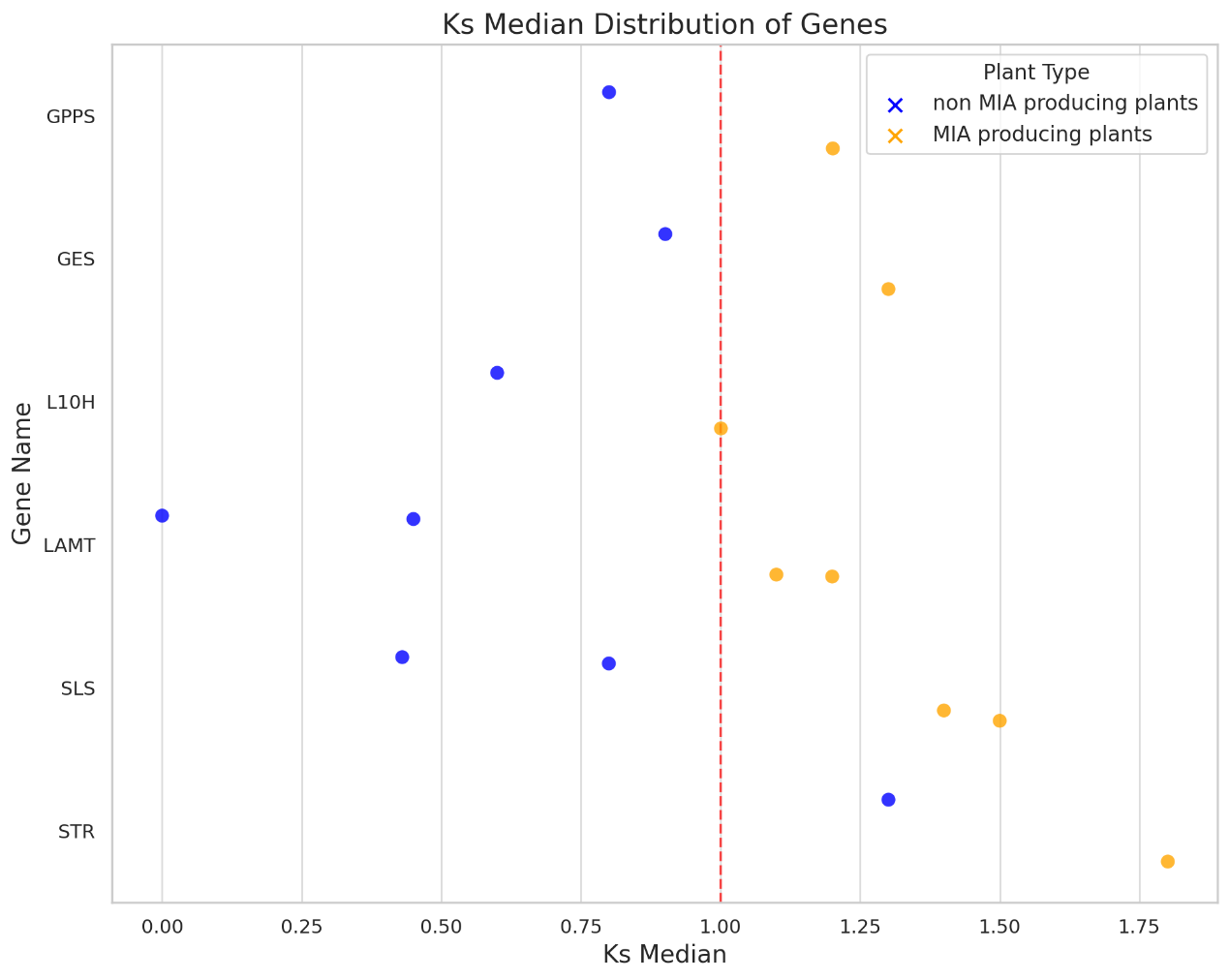
**Figure. S14. Median synonymous substitutions per synonymous sites (*Ks*) distribution for genes associated with loganin biosynthesis pathways across MIA plants.** Dotted red line refers to *Ks* median for functional *LAMT* in genome. Orthogenes highlighted by red color are the orthogenes specifically gained or expanded in MIA plants.

**
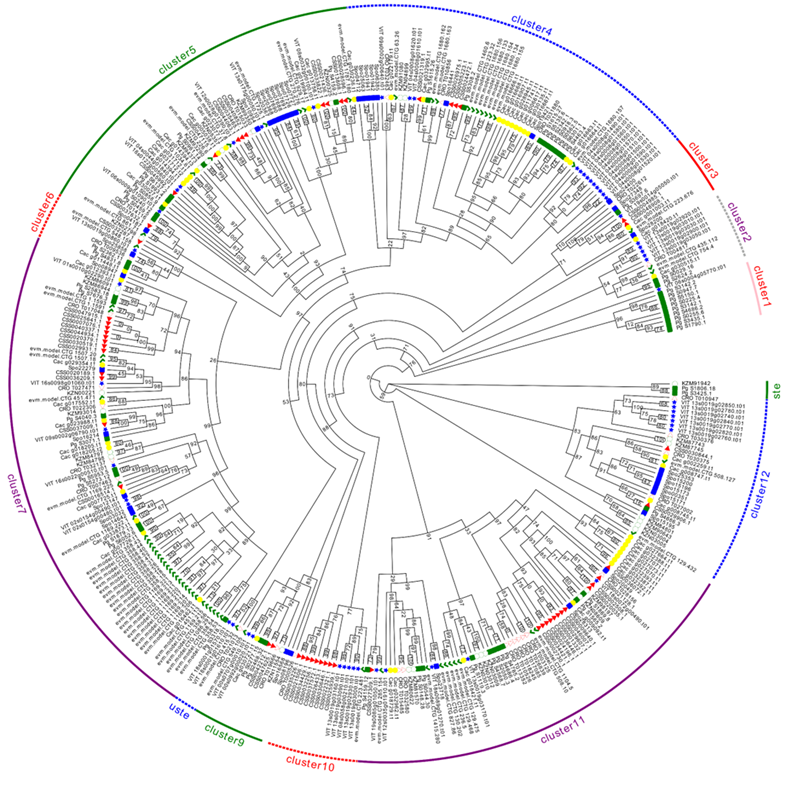
**

**Figure. S15.** **Phylogenetic tree of the candidate *LAMTs* and *SLSs* encoding genes identified in *C. officinalis* and seven MIA plants.** Different colors indicate different *LAMTs* and SLSs members classified based on the protein domain annotation.


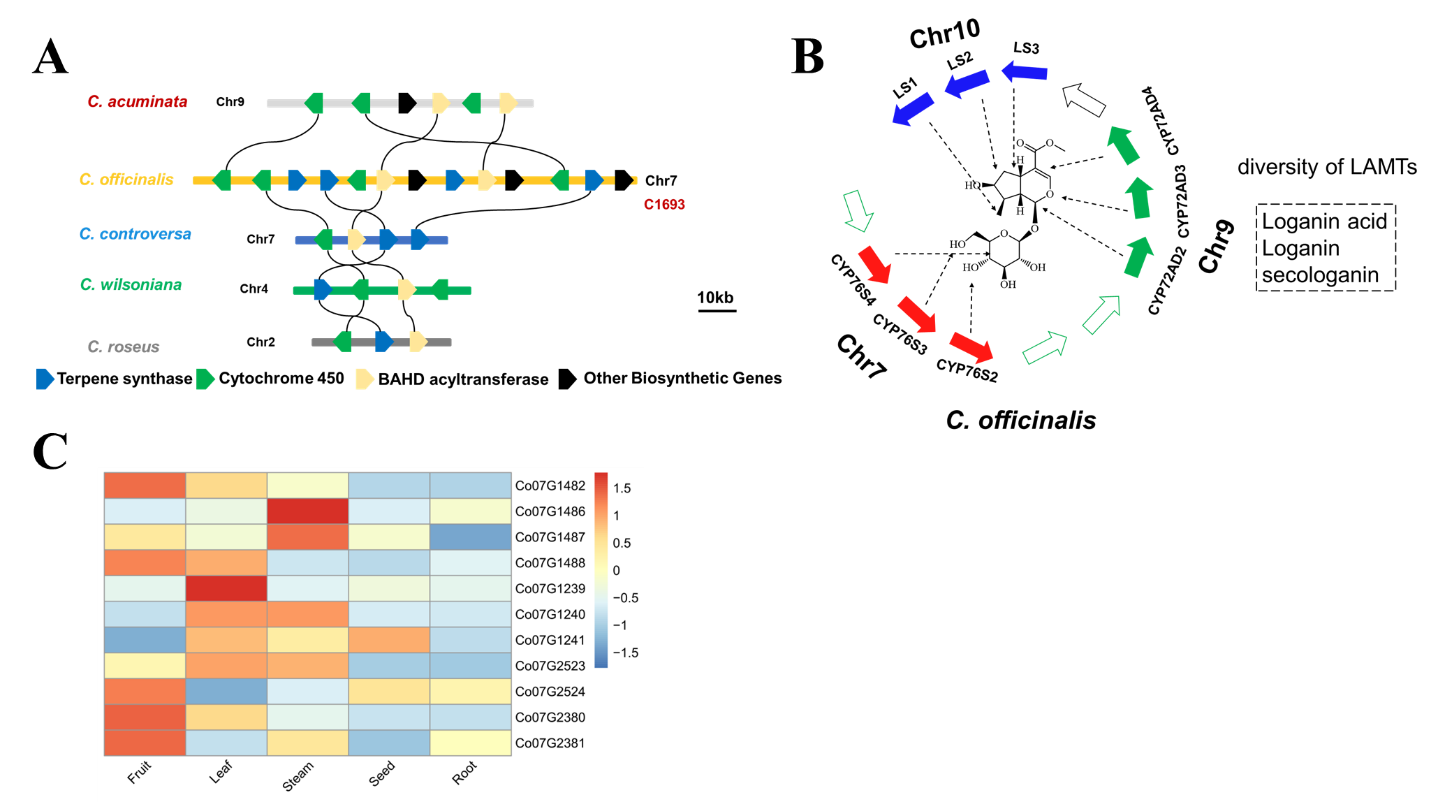
**Figure. S16. The evolution of monoterpene loganin biosynthesis in plants. A.** Conserved gene clusters, C1693, essential for the biosynthesis of loganin. Dashed lines show syntenic blocks, while scaffolds for each genome are shown through distinct color. **B.** This diagram illustrates the directional flow of substrate conversion catalyzed by various enzymes. The arrows represent the different catalytic steps, with green arrows indicating forward reactions, red arrows representing reverse reactions, and blue arrows depicting the substrate binding and release process. The structure highlights the sequential events in the enzyme's action, with particular attention to the directionality of the metabolic pathways involved. **C.** Expression profiles for the nine C1693 genes in *C. officinalis*.


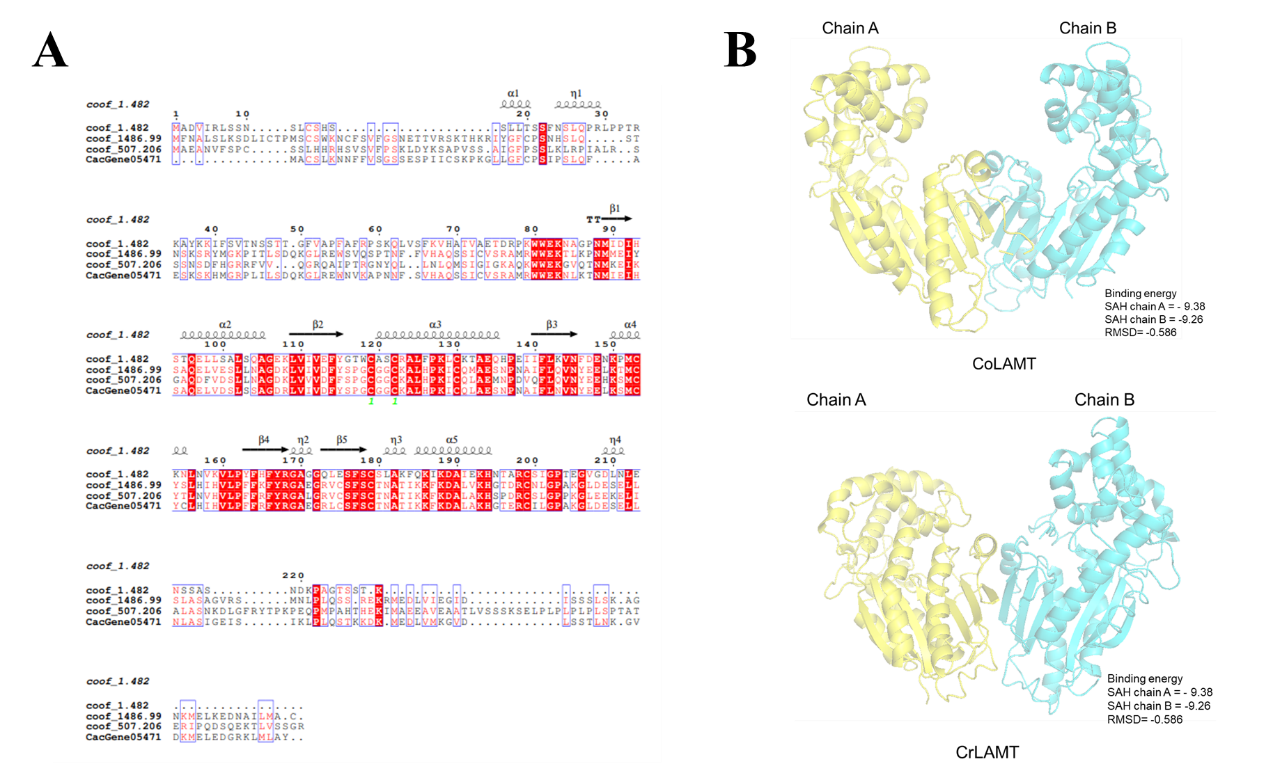
**Figure. S17. Alignment of amino acid sequence of *LAMT* encoding gene copies in *C. officinalis* and other species. A.** Maximum likelihood tree of the transcripts homologous to LAMT. **B.** Phylogenetic relationship and protein sequence alignment of LAMT homologs from other species.


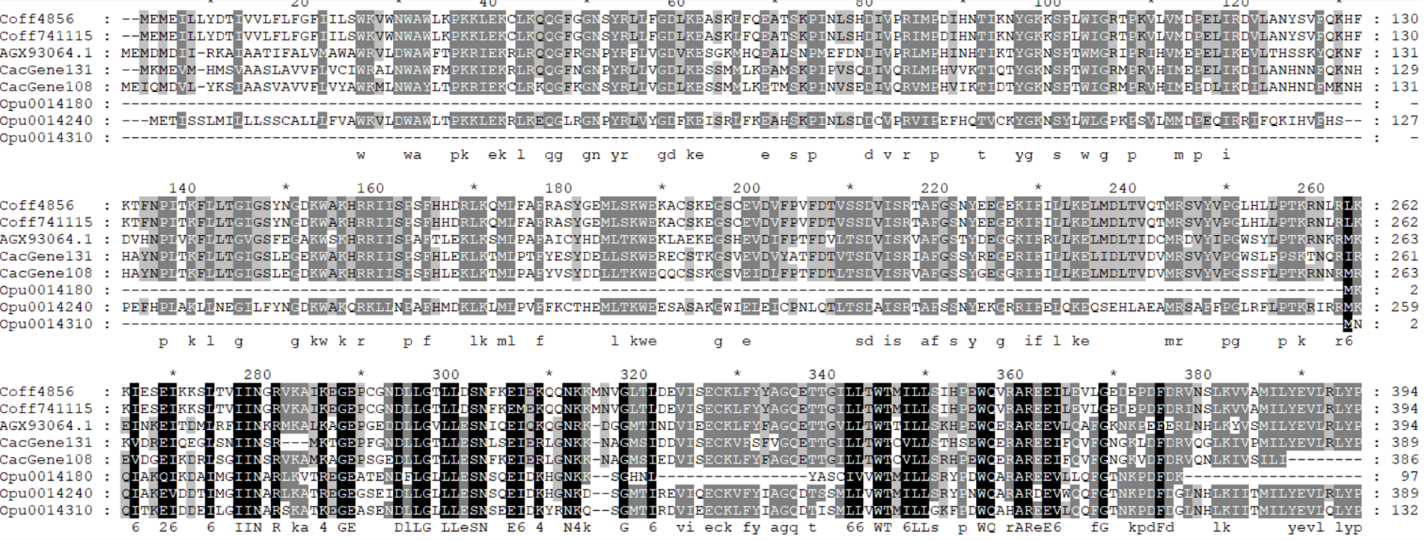
**Figure. S18. Alignment of amino acid sequence of SLS encoding gene copies in *C. officinalis* and other species.**

**
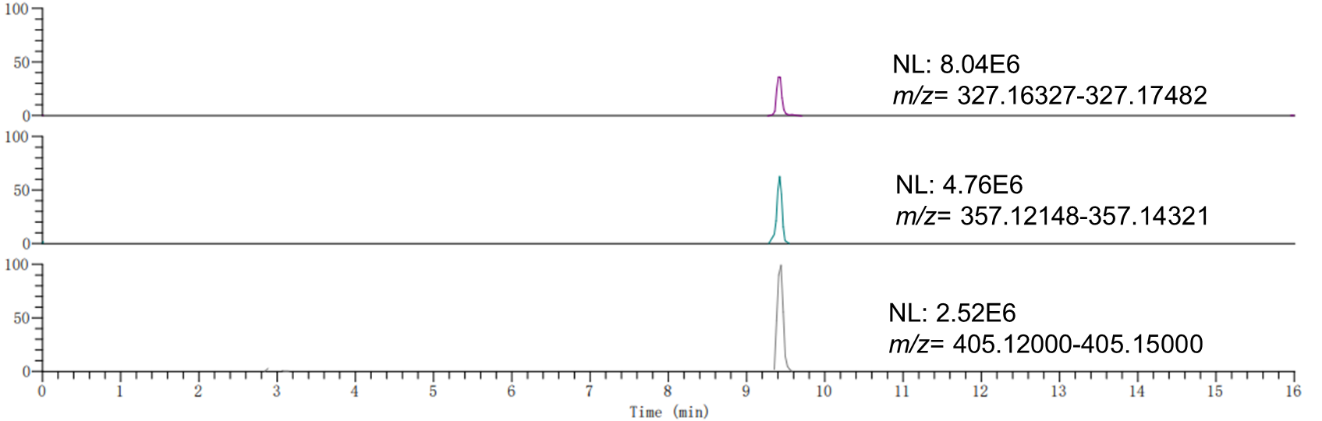
Figure. S19. EIC for 9-loganin in *N. benthamiana* leaves expressing Co1.482, Co507.206, and Co1486.99, with the infiltration of CoLAMT.** The experiment was repeated independently three times with similar results.


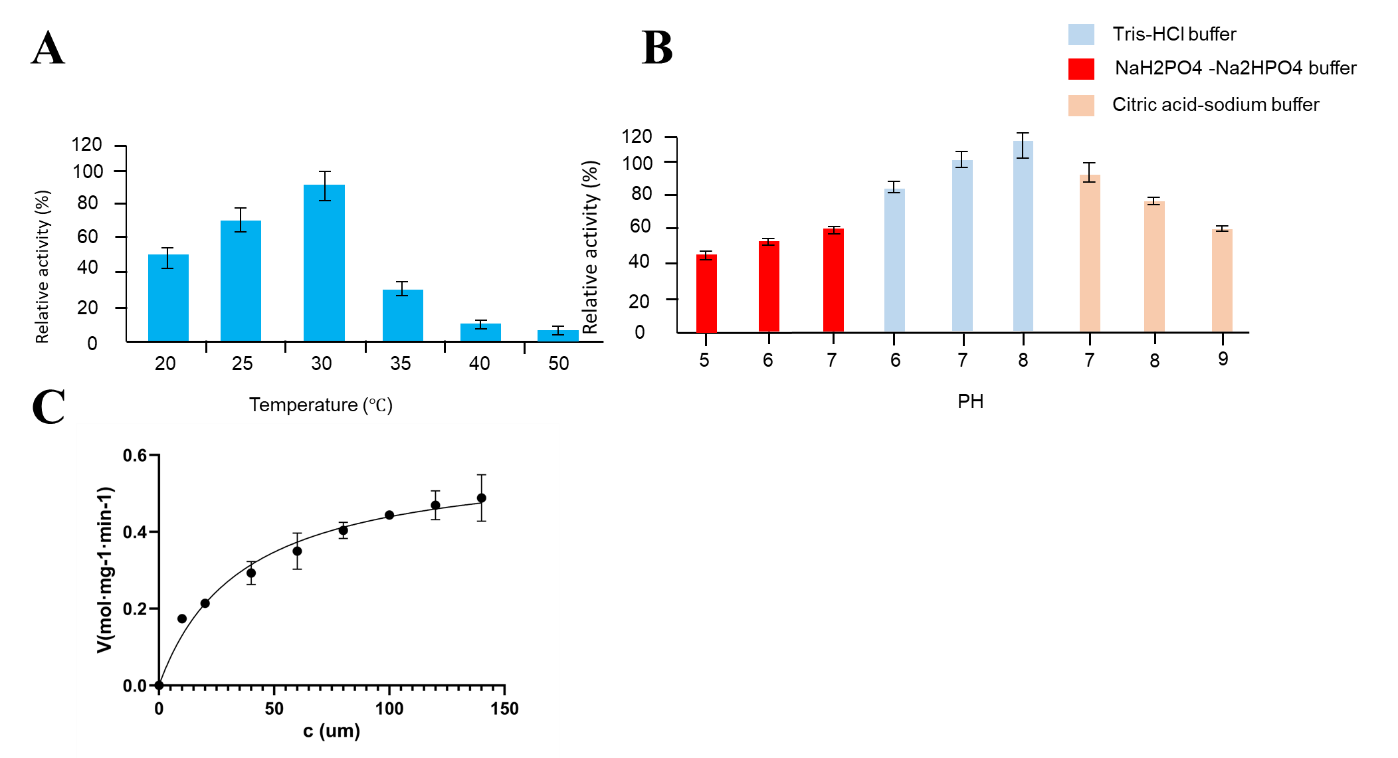
**Figure. S20. Biochemical properties of Co 1.482. A.** Enzymatic activity of Co 1.482 at different temperatures. **B.** Enzymatic activity of Co 1.482 with buffer solutions of different pH values. **C.** Kinetic parameter of Co 1.482 with loganin as substrate.

**
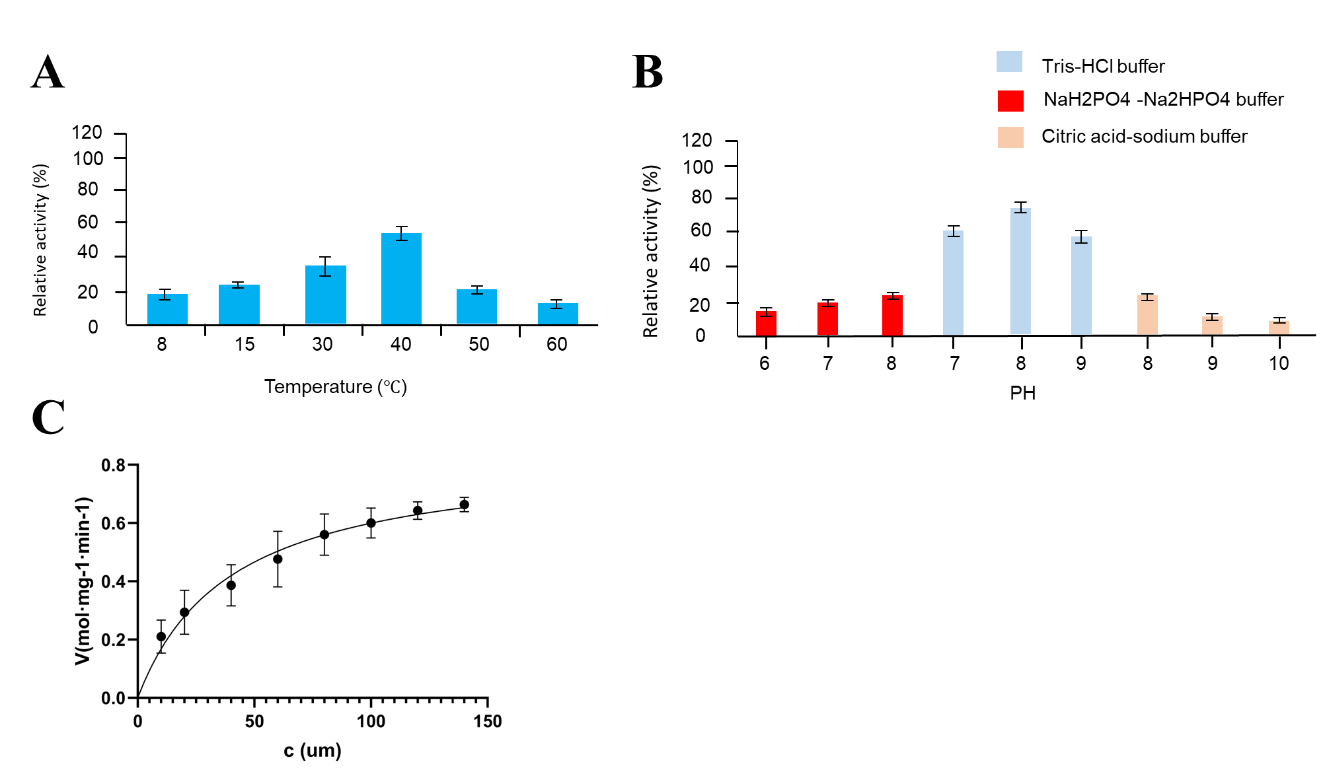
**

**Figure. S21. Biochemical properties of Co 507.206. A.** Enzymatic activity of Co507.206 at different temperatures. **B.** Enzymatic activity of Co507.206 with buffer solutions of different pH values. **C.** Kinetic parameter of Co507.206 with loganin as substrate.


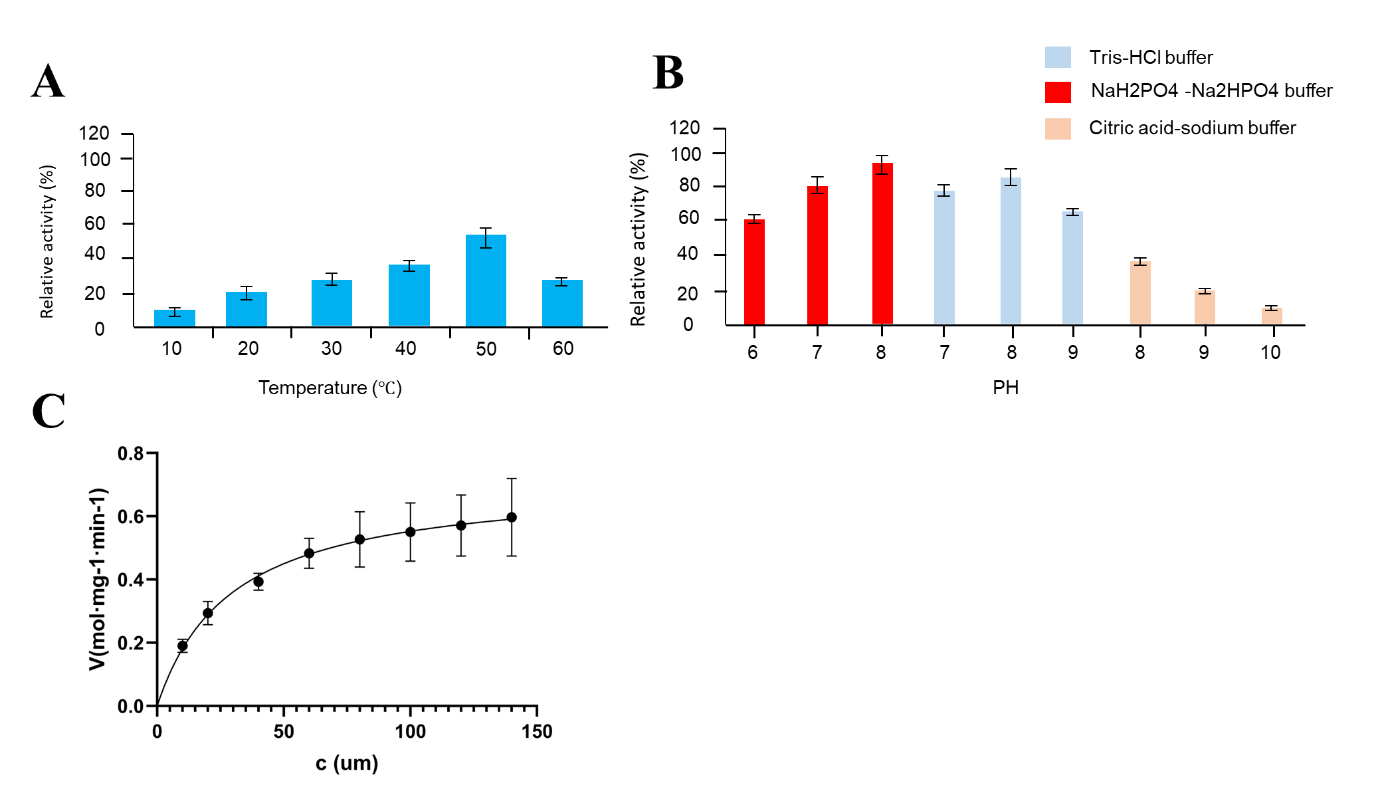
**Figure. S22. Biochemical properties of Co1486.99. A.** Enzymatic activity of Co1486.99 at different temperatures. **B.** Enzymatic activity of Co1486.99 with buffer solutions of different pH values. **C.** Kinetic parameter of Co1486.99 with loganin as substrate.


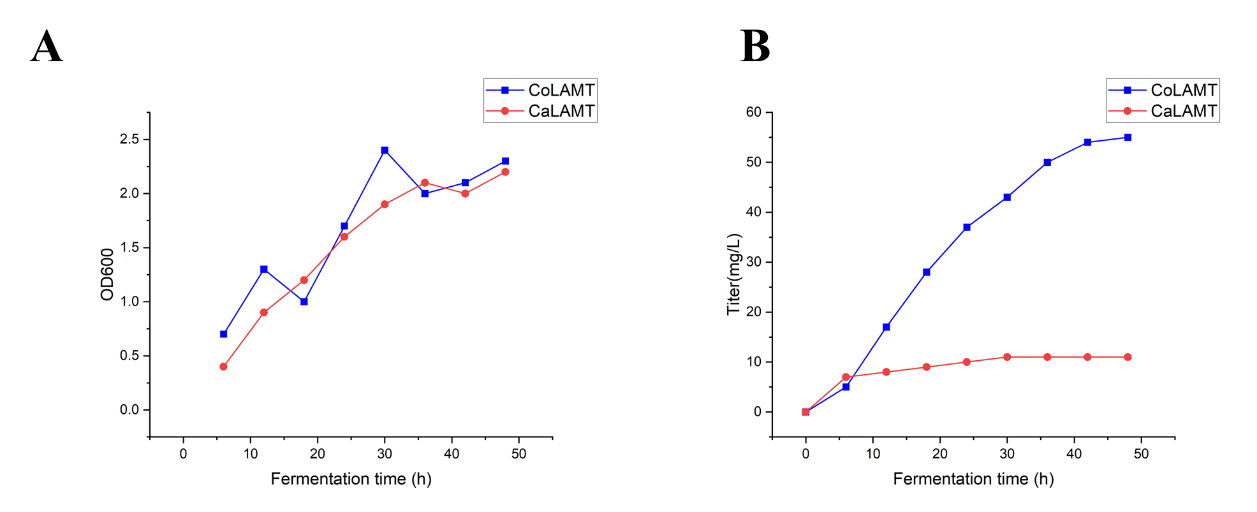


**Figure. S23. Comparison of the two types of LAMT on the production of loganin A.** The cell concentrations of the strains CoLAMT and CaLAMT were measured by OD600 at set intervals (at 0, 6,12,18,24,32,36,40 and 48 hours) **B**. The titers of loganin produced by CoLAMT and CaLAMT in shaking flasks.


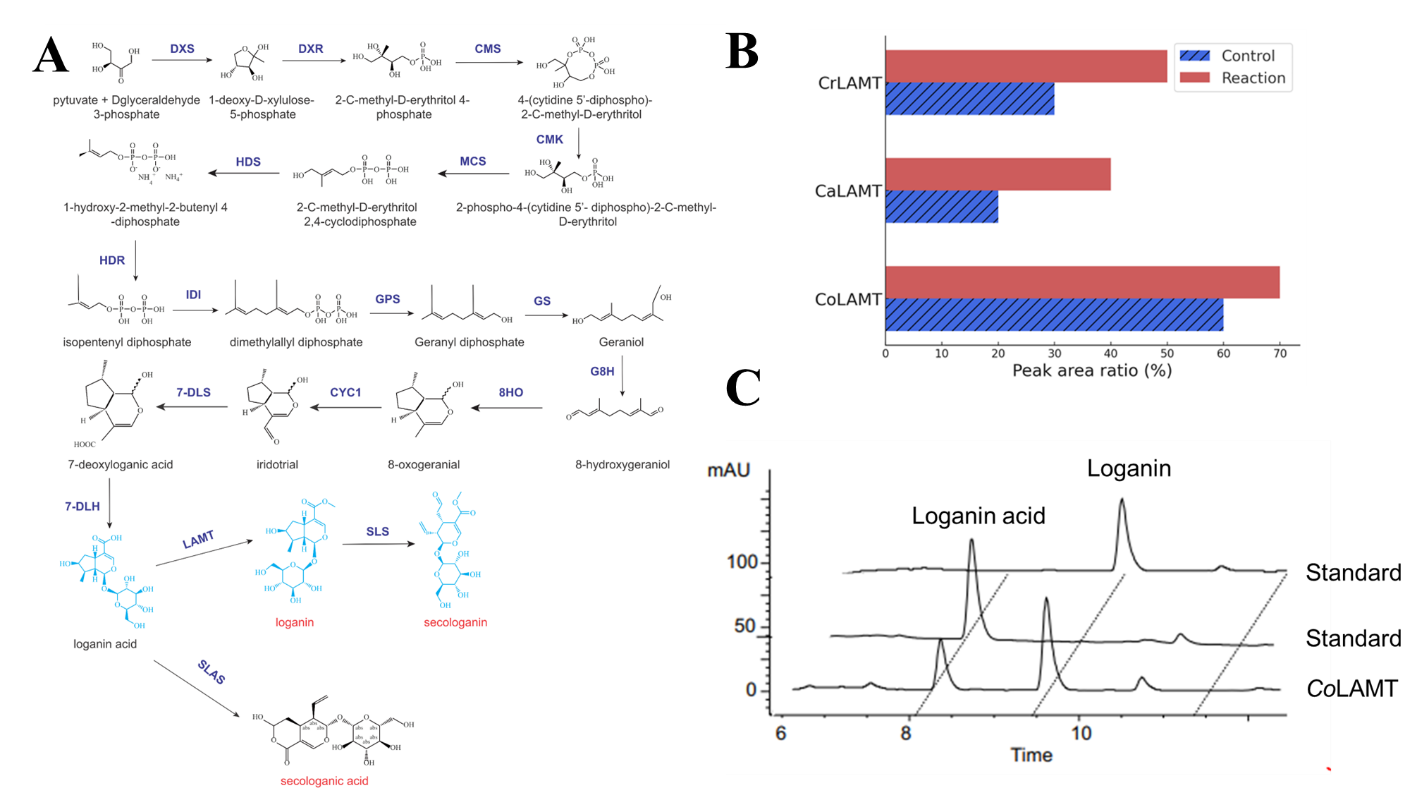
**Figure. S24. Identiﬁcation of *LAMT* biosynthetic genes in *C. officinalis* A.** The partial biosynthesis pathway of loganin. A representative result of n = 3 independent experiments is shown. **B**. A comparison of the peak area ratios between the control and reaction groups for each enzyme is shown using bar charts, illustrating the differences between the groups. **C.** A comparison of the peak area ratios between the control and reaction groups for each enzyme is shown using bar charts, illustrating the differences between the groups.


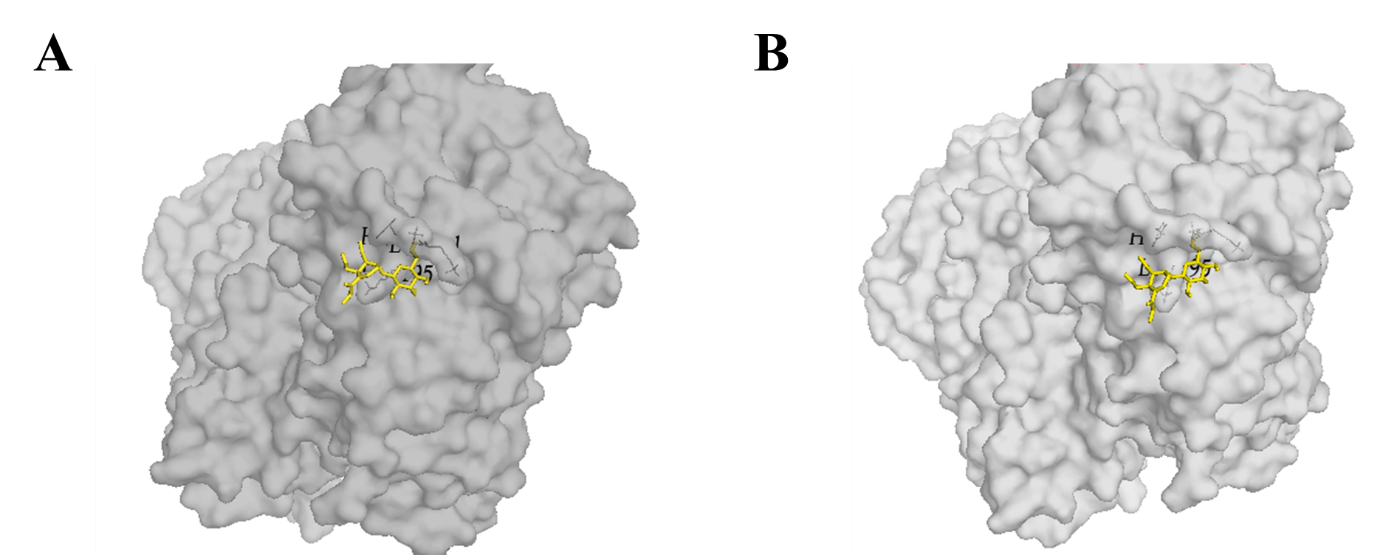
**Figure. S25. The surface presentation of CoLAMT and CaLAMT.**  **A.** The surface presentation of CoLAMT showed the inner tunnels. Hydrogen bonds and hydrophobic interactions were shown as yellow dashed lines. **B.** The surface presentation of CaLAMT showed the inner tunnels. Hydrogen bonds and hydrophobic interactions were shown as yellow dashed lines.
