# Supplementary material for: Chromosome-level genome assembly of Cornus officinalis reveals the evolution of loganin biosynthesis
Source: Hortic Res. 2025 Sep 24;13(1):uhaf259. doi: 10.1093/hr/uhaf259 (PMC12860560; doi:10.1093/hr/uhaf259)
Supplement: Web_Material_uhaf259 [file web_material_uhaf259.zip › Supplementary methods 1-13.docx]

**Supplementary methods**

**Method S1.** Evaluation of genome size and heterozygosity.

**Method S2.** RNA-seq sequencing.

**Method S3.** *De novo* genome assembly and assessment.

**Method S4.** Repeat annotation.

**Method S5.** Gene prediction and function annotation.

**Method S6.** Phylogenetic analysis.

**Method S7.** Gene expression analysis.

**Method S8.** WGD events analysis.

**Method S9.** Phylogenetic analysis of TPS- and P450-encoding genes.

**Method S10.** Transcription factors analysis.

**Method S11** Intron and Exon Expression Quantification

**Method S12.** Whole-genome bisulfite sequencing (WGBS).

**Method S13.** Metabolic gene cluster prediction.

**Supplementary methods**

**Method S1.** Evaluation of genome size and heterozygosity

We obtained fresh leaves and determined their genome size using an Elite flow cytometer (MoFlo, XDP, USA). To extract the nucleus from young leaves, we used a wood plant buffer. We then filtered the nuclear suspension with 300-mesh nylon mesh and stained it with 50 μg mL^-1^ of propidium iodide (PI) (<https://kns.cnki.net/kcms/detail/46.1068.S.20211103.1023.004.html>). After staining the nucleus at room temperature for 10 minutes, we measured the fluorescence value using the MoFlo XDP flow cytometer.

To identify the genome size of *C. officinalis*, we selected one plant of known genome size (*Zea mays*, 2.18 Gb) as an internal standard on a flow cytometric analyzer. We calculated the genome size using the formula N = (P-sample/P-target) × C and estimated the average genome size for *C. officinalis* to be 2.74 Gb.

We utilized the standard *K*-mer analysis approach to estimate the genomic features and genome size of *C. officinalis*. A total of 73,532,815,926 *K*-mers were identified using Jellyfish (version 2), with the highest peak observed at a depth of 39. Based on these results, we estimated the size of the *C. officinalis* genome to be approximately 2.97 Gb using the equation G = *K*-mer-number/*K*-mer depth. The fitted *K*-mer curves indicated that the proportion of heterozygous and repetitive sequences were estimated to be 0.39% and 80.39%, respectively.

**Method S2.** RNA-seq sequencing

To perform RNA-seq analysis, we collected fresh tissues from five different parts of the same tree: young leaves, old leaves, roots, seeds, and fruits. The tissues were immediately stored in liquid nitrogen and kept at -80 ℃ until RNA extraction. In total, we constructed 15 sequencing libraries using the RNAprep Pure Plant Kit (TIANGEN Biotech Co., Ltd., Beijing, China, Cat No. DP432), with a target insert length of 200-300 bp. Finally, we performed 150 bp paired-end sequencing on an Illumina platform, generating a total of 309.8 G raw reads.

**Method S3.** *De* *novo* genome assembly and assessment

To determine the assembly strategy for this study, we conducted a preliminary Genome Survey analysis. Based on the results, we utilized a de novo genome assembly approach that involved contig-level assembly using Oxford Nanopore Technologies and chromosome-level assembly using Bionano mapping and Hi-C scaffolding. First, we assembled long sequence reads generated by Nanopore sequencing using SMARTdenovo [^1^](#_ENREF_1) with default parameters to identify completely covered gaps [^2^](#_ENREF_2). Next, we used optical molecules with a length greater than 180 kb or with a molecule label number greater than 9 to perform optical map assembly using the Bionano Solve Pipeline v.3.3 (<https://bionanogenomics.com/support/software-downloads/>). We generated hybrid scaffolds by aligning the optical maps to Nanopore-assembled genomic contigs using Bionano's hybrid-scaffold software. Finally, we aligned Hi-C data to the reference genome scaffolds using the 3D-DNA pipeline (version 180922) (<https://github.com/aidenlab/3d-dna>) software. We manually corrected several misassembled scaffolds using the Juicer pipeline (v1.8) (<https://github.com/aidenlab/Juicer>). After scaffold adjustment, assembled contigs and scaffolds were clustered, reordered and oriented to the chromosome level by the LAchesis software [^3^](#_ENREF_3). The Hi-C contact maps were visualized using Juicer Tools (<https://github.com/aidenlab/Juicebox/releases>). In total, we generated an assembly with an N50 of 4.42 Mb and the total length of 2,863.50 Mb.

We estimated the completeness and quality of the *C. officinalis* genome assembly using five different assessments. First, we calculated the mapping rates of the clean reads from RNA-seq data of five tissues (young leaves, old leaves, root, fruits, and seeds) by TopHat2 v2.1.0 (<https://github.com/infphilo/hisat2>) with default parameters. Second, we utilized the LTR Assembly Index (LAI) [^4^](#_ENREF_4) to evaluate the assembly continuity. Third, 95.16% of completed genes were identified through Benchmarking Universal Single-Copy (BUSCO) v2.0 [^5^](#_ENREF_5) assessment. Fourth, BWA-MEM (<https://github.com/lh3/bwa>) was used to map the clean reads to the assembly, with the mapping rate of 99.65% and a high confidence coverage of 97.57%. Finally, a high sequence coverage of 98.7% was obtained by mapping Nanopore long reads to the assembly using minimap2 (version 2.11) [^6^](#_ENREF_6).

**Method S4.** Repeat annotation

To annotate repeat sequences in the *C. officinalis* genome, we combined the application of RepeatModeler v1.0.10 (<http://www.repeatmasker.org/RepeatModeler/>) and RepeatMasker v 4.0 (<http://www.repeatmasker.org>) [^7^](#_ENREF_7) to predict and annotate repeat elements. RepeatModeler was used to identify repetitive elements based on *de novo* prediction. RepeatMasker was applied to perform a homology-based annotation search of the *C. officinalis* genome using both the *de novo* repeat database and the ab initio repeat database from RepBase (<http://www.girinst.org/repbase/>)[^8^](#_ENREF_8).

To identify long terminal repeat retrotransposons (LTR-RTs), we initially used LTR_Finder v1.02 [^9^](#_ENREF_9) and LTRharvest [^10^](#_ENREF_10). The superfamily classification of transposon elements was estimated through LTR_finder. LTRharvest was used to separate the candidate LTR sequences of *C. officinalis* with parameters: -minlenltr 100 -maxlenltr 7000 -mintsd 4 -maxtsd 6 -motif TGCA -seqids yes. The BLASTN algorithm (version 2.9.0) was used to reciprocally compare the full-length LTR of each enriched LTR subfamily in *C. officinalis* and LTR in other species. In this analysis, we used *E*-value threshold of < 1 × 10 ^-30^, identity > 80%, and query coverage > 75% to define homologous TEs in *C. officinalis*.

LTR_retriever [^11^](#_ENREF_11) was used to merge the results of the two runs and obtain high confidence LTR-RT sequences with parameters: -in harvest specie. LTR.scn -threads 10 -u 1e-8. We further examined the insertion time (T) of LTR-RTs. The insertion time (T) of LTRs was calculated with the following formula T = *d/2u*, where *u* is the *Arabidopsis thaliana* neutral mutation rate (*u* = 1.5 ×10 -8) [^3^](#_ENREF_3)^,^[^12^](#_ENREF_12) and *d* is the divergence time. ClustalW (version 1.81) (<http://www.clustal.org/>) was applied to search 30 and 15 solo-LTRs corresponding to intact LTR elements. The evolutionary divergence between the two LTR sequences was assessed by the Kimura two parameter approach, which was incorporated within BASEML in PAML [^13^](#_ENREF_13) (<http://abacus.gene.ucl.ac.uk/software/paml.html>). To infer the insertion age of retrotransposons from the evolutionary distance between the 30 and 15 solo-LTRs, a substitution rate of 1.3×10^-8^ mutations per site per year was employed.[^3^](#_ENREF_3)

**Method S5.** Gene prediction and function annotation

Gene structure prediction was performed using the comprehensive strategies of *de novo* prediction, homology-based annotation and RNA-seq-based pipelines. For the *de novo* prediction, SNAP [^14^](#_ENREF_14), Augustus v3.0.2 [^15^](#_ENREF_15) and GlimmerHMM (v3.0.1) were applied. Five species (*A. thaliana* [^16^](#_ENREF_16), *P. ginseng*, *D. carota*, *C. roseus,* and *P. trichocarpa*) were selected for homology annotation to predict protein-coding genes using GeneWise. In order to support genome annotation, we also mapped RNA-seq data to the reference genome assembly using HISAT2 with default parameters. Finally, the results from the three approaches were integrated for correction and redundancy removal using the EVidenceModeler (EVM) to obtain the final annotated protein-coding gene set.

We annotated non-coding RNAs (ncRNAs) using several databases and software packages. First, tRNAs and their secondary structures were annotated using tRNAscan-SE (version 1.3.1) with default parameters. Second, ribosomal RNAs (rRNAs) were annotated using RNAMMER (version 1.2). Finally, we searched the Rfam database (version 9.1) (<http://eggnogdb.embl.de/>) using BLASTN to annotate other ncRNAs.

After the structural annotations, we also annotated the functions of the predicted protein-coding genes. The putative functions of the genes were predicted by searching the best-matched proteins in the SwissProt (<https://web.expasy.org/docs/swiss-prot_guideline>), non-redundant (Nr) (https://ftp.ncbi.nlm.nih.gov/blast/db/FASTA/) and Eukaryotic Orthologous Groups (KOG) (<https://hsls.pitt.edu/obrc/index.php?page=URL1144075392>) databases using BLASTP (E-value ≤ 10 -10). Gene ontology [^17^](#_ENREF_17) terms were assigned to the genes by BLAST searches against the Gene Ontology (GO) database (<http://geneontology.org/>). We also used the KEGG database (https://www.genome.jp/kegg/) to obtain KEGG orthologues to infer putative gene pathways.

By integrating all strategies for gene function annotations, 95.5% of all predicted genes could be annotated with the following outcomes for at least one of the protein-related databases: InterPro [^18^](#_ENREF_18) (90.40%), Nr (94.80%), Pfam (71.40%) [^17^](#_ENREF_17), SwissProt (75.20%), KEGG (78.60%) and GO (51.90%).

**Method S6.** Phylogenetic analysis

To verify the phylogenetic position of *C. officinalis*, seven representative plant species (*C. sinensis*, *D. carota, C. acuminata*, *C. roseus*, *P. ginseng*, *S. oleracea* and *V. vinifera*) were identified using OrthoFinder v2.3.1 [^19^](#_ENREF_19) with parameters -t 20 -a 20. To minimize the alternative splicing variants of genes influence, we selected the longest transcript to represent the gene in each species. Protein sequences were aligned using MUSCLE v3.8.31 with default settings. GBlocks was used to trim alignments. Based on the trimmed alignments, we constructed a maximum likelihood (ML) tree using IQ-TREE version 1.6.7 with the JTT+F+I+G4 models, Shimodaira-Hasegawa-like approximate likelihood-ratio test (SH-aLRT >= 80 %) and *V. vinifera* as an outgroup. To estimate the divergence times among the eight species, MCMCtree program in the PAML package (version 4.9j) were used to calculate with the parameters: burnin = 2000, sampfreq = 10, nsample = 20,000. Fossils calibration times were determined using the TimeTree website (<http://www.timetree.org/>). Finally, the phylogenetic tree was visualized using the R package GGTREE [^20^](#_ENREF_20).

**Method S7.** Gene expression analysis

Differential gene expression (DEG) analyses among the five tissues were performed using DEseq2 [^21^](#_ENREF_21) and with an FDR cut-off of 0.05 and log_2_ fold change (FC) cut-off of 1. GO enrichment analysis of DEGs was performed using the GOseq R package, which corrects for gene length bias. KOBAS software was used to test the enrichment of DEGs in KEGG pathways.

**Method S8. Whole-genome duplication (**WGD) events analysis

To identify the WGD events, we analyzed the distribution of Ks values for each paralog in *C. officinalis.* Homologous gene pairs of proteins were identified using an all-against-all BLASTP search with an *E*-value cutoff of 1e-10. MCScanX [^22^](#_ENREF_22) with parameters “-E 1e-5 -m 25 -w 5” was used to detect syntenic blocks between *C. officinalis*, *D. carota,* and *V. vinifera,* as well as within each species, each block containing at least five homologous gene pairs. Ks peaks of *C. officinalis*, *D. carota,* and *V. vinifera* syntenic orthologs were calculated the equation: time = Ks/2u, where u is the synonymous substitution rate per site per year, estimated at 8.6 × 10^-9^. Genes were further classified by DupGen_finder [^23^](#_ENREF_23) (<https://github.com/qiao-xin/DupGen_finder>) with default parameters. The Ka (substitutions per nonsynonymous site), Ks, and Ka/Ks values were computed for gene pairs resulting from various duplication modes using the YN model within KaKs_Calculator.

**Method S9.** Phylogenetic analysis of TPS- and P450-encoding genes

The initial steps involved in retrieving Hidden Markov Model (HMM) files for the two conserved domains PF05860 of the TPS gene family, as well as PF00067 of the P450 gene family, from the Pfam database. The genes were then amalgamated with the HMM files and subsequently searched against the *C. officinalis* genome using HMMER. The results from both BLAST and HMMER detection were merged, and genes lacking conserved functional structural domains were excluded. The TBtools Gtf/Gff3 sequence extractor utility was employed to extract a 2000 bp promoter sequence upstream of these genes. The promoter sequences were evaluated using the web-based online tool PlantCARE (<http://bioinformatics.psb.ugent.be/webtools/plantcare/html/>) to analyze promoter sequence binding elements. For waxberry TPS/P450 gene structure analysis, the GSDS 2.0 online tool (<http://gsds.cbi.pku.edu.cn/index.php/>) was utilized. Phylogenetic trees of DEGs and genes of other species obtained from NCBI were constructed using MEGA X [^24^](#_ENREF_24).

Characterization of the protein sequences revealed conservation of an N-terminal cytochrome b5-like domain and three histidine-rich boxes in TgDES1 clade desaturases (clade 1) and their two closely related groups. Phylogenetic trees constructed form DEGs and homologous genes from other species (obtained from homology database) indicated that these genes exhibit high expression levels in different tissues. Divergence times between *C. offinicinalis* and other species were estimated based on the phylogenetic tree constructed using protein sequences containing conserved binding elements.

**Method S10.** Transcription factors analysis

Transcription factors (TFs) were identified using iTAK (<http://itak.feilab.net/cgi-bin/itak/index.cgi>) based on homology to *A. thaliana*. We also used the STEM (<http://www.cs.cmu.edu/~jernst/stem/>) software for differential expression analysis. HMM profiles were downloaded from Pfam and used as queries to search against protein sequence databases using the HMMER software (<http://hmmer.org/>), with E-value thresholds set to 1e-10. Furthermore, all obtained protein sequences were manually inspected with SMART (<http://smart.embl-heidelberg.de/>) to verify the presence of conserved domains. The NCBI Batch Web CD-Search Tool (<https://www.ncbi.nlm.nih.gov/Structure/bwrpsb/bwrpsb.cgi>) was used to confirm domains presence, employing default parameters. The conserved motifs and structures identified for each TF family were predicted by TBtools (version 0.6644449).

**Method S11** Intron and exon expression quantification

To quantify the expression levels of intron regions, first introns, and intron-less exonic regions, we used strand-specific, high-depth RNA-seq data. Reads were aligned to the reference genome using STAR (v2.7) with default splice-aware parameters, and only uniquely mapped reads were retained. Gene annotation was obtained from the curated genome release (version X.X) and refined via StringTie2-guided transcript assembly.

Intron regions were defined based on the genomic intervals between annotated exons of multi-exon genes. First introns were identified as the intron immediately downstream of the first exon. Intron-less regions were selected from single-exon genes, excluding transposon-related or pseudogene sequences based on Pfam and RepeatMasker filtering.

Read counts overlapping these regions were obtained using featureCounts (Subread v2.0.1), with a minimum overlap threshold of 10 bp. Regions with total read counts <10 across all samples were excluded. Normalized expression values were calculated using TPM (Transcripts Per Million) to adjust for differences in gene length and sequencing depth. Quality control steps included the exclusion of genes with >20% intron retention and removal of ambiguous alternative splicing regions.

**Method S12.** Whole-genome bisulfite sequencing (WGBS)

A Bioruptor system was used to combine 1 µg of genomic DNA sample with unmethylated lambda DNA, then fragment it to an average size of approximately 250 bp. The purified, randomly fragmented DNA was then repaired, blunt-ended, and phosphorylated using a mixture of T4 DNA polymerase, Klenow fragment, and T4 polynucleotide kinase. Libraries were constructed through end repair, A-tailing, adapter ligation, and bisulfate-conversion using a ZymoEZ DNA methylation kit (Zymo Research, CA, USA). The insert size of the library was evaluated using Agilent 2100. After confirming that the insert size met expectations, RT-qPCR was used to accurately quantify the effective concentration of the library ( > 2 nM) to ensure the quality.

After sequencing on an Illumina platform with PE150, the libraries were filtered using Trim_galore. The filtered reads were then mapped to the reference genome using Bismark (v0.14.3) with default parameters. To calculate whole-genome methylation level, we used BatMeth2 [^25^](#_ENREF_25) with parameter -Q 20 --remove_dup --coverage 4 -nC 1 --Regions 600 --step 50000. The ‘methylKit’ R package (v0.9.5) with default parameters was used to differential methylation sites (DMSs) and differentially methylated regions (DMRs) with a P value of < 0.05. For gene and TE methylation level analysis, the region from 2,000 bp upstream to 2,000 bp downstream of the transcription start site (TSS) was defined as the promoter, and this criterion was applied in PeakAnnotator (v1.4) with default parameters. To determine the correlation between DNA methylation and kimura distance, the DNA methylation level of each TE was computed using a custom Perl script.

**Method S13.** Metabolic gene cluster prediction

For gene cluster analysis, we used PlantiSMASH software (v 1.3). *C. officinalis* gene models were assigned four-part EC numbers and MetaCyc reaction identifiers based on protein sequence data and classification according to the predicted catalytic functions by E2P2 software (v 3.1)141. E2P2-based enzyme annotation assigned *C. officinalis* genes with EC numbers, which were then converted into the corresponding MetaCyc (v 22.5) reaction identifiers. These identifiers were used for pathway inference and pathway database construction using PathoLogic software (v 22.5) in the Pathway Tool software. The derived pathways database was then manually curated and validated, using SAVI software (v 3.0.2) to remove any false positive and redundant pathways, such as non-plant pathway variants, and pathways already included as part of a larger pathway. The pathway database for *O. pumila* with assigned metabolic reactions and enzymes was then used as input, together with *C. officinalis* genome annotation, for the PlantClusterFinder software, as instructed by the tool developers. Using the PlantClusterFinder pipeline, we identified 358 gene clusters representing 3,387 genes of the *C. officinalis* genome. To assign gene clusters to MIA biosynthesis, we mapped the *C. officinalis* genome annotation using the MIA protein database and considered a gene cluster as an MIA gene cluster if it included one or more of the 216 high-confidence MIAs biosynthesis genes. The obtained gene clusters were mapped with synteny data to compare the *C. officinalis* genome with three other MIA-producing plants, namely, *C. acuminata,* *C. roseus*, and *G. sempervirens*. Despite the fragmented genome assemblies of the other MIA-producing plants, synteny analysis showed a conserved gene order centered on *C. officinalis* MIA gene clusters, which was statistically significant based on a one-sided Fisher exact test.

# References:

1. Schmidt, H.W., Vogel, A., Denton, A., Istace, B. & Usadel, B. Reconstructing the Gigabase plant genome of *Solanum pennellii* using Nanopore Sequencing. *Cold Spring Harbor Labs Journals* (2017).

2. Li, Y.L. et al. A protocol of homozygous haploid callus induction from endosperm of *Taxus chinensis* Rehd. var. mairei. *Springerplus* **5,** 659 (2016).

3. The sunflower genome provides insights into oil metabolism, flowering and Asterid evolution. *Nature* **546(7656)**, 148-152 (2017).

4. Shujun, Chen, Jinfeng, Jiang & Ning. Assessing genome assembly quality using the LTR Assembly Index (LAI) *Nucleic acids research*, **46(21)**, e126-e126 (2018).

5. Simão, F., Waterhouse, R.M., Panagiotis, I., Kriventseva, E.V. & Zdobnov, E.M. BUSCO: assessing genome assembly and annotation completeness with single-copy orthologs. *Bioinformatics* **31(19)**, 3210-3212 (2015).

6. Heng. Minimap2: pairwise alignment for nucleotide sequences. *Bioinformatics* **34(18),** 3094-3100 (2018).

7. Tarailo‐Graovac, M. & Chen, N. Using RepeatMasker to Identify Repetitive Elements in Genomic Sequences. *Current Protocols in Bioinformatics* 25, **5(1),** 4-10 (2009).

8. Jurka, J. Repbase update: a database and an electronic journal of repetitive elements. *Trends in Genetics* Tig **16,** 418-420 (2000).

9. Zhao, X. & Hao, W. LTR_FINDER: an efficient tool for the prediction of full-length LTR retrotransposons. *Nucleic Acids Research* **35,** W265-8 (2007).

10. Ellinghaus, D., Kurtz, S. & Willhoeft, U. LTRharvest, an efficient and flexible software for *de novo* detection of LTR retrotransposons. *BMC Bioinformatics* **9,** 1-14 (2008).

11. Ou, S. & Ning, J. LTR_retriever: A highly accurate and sensitive program for identification of long terminal repeat retrotransposons. *Plant Physiology* **176,** 01310 (2017).

12. Koch, M.A., Haubold, B. & Mitchell-Olds, T. Comparative evolutionary analysis of chalcone synthase and alcohol dehydrogenase loci in *Arabidopsis*, Arabis, and related genera (*Brassicaceae*). *Mol Biol Evol* **17,** 1483-98 (2000).

13. Ziheng, Y. PAML 4: Phylogenetic Analysis by Maximum Likelihood. *Molecular Biology and Evolution* **24,** 1586-1591 (2007).

14. Ismail, W.M., Ye, Y. & Tang, H.J.B.B. Gene finding in metatranscriptomic sequences **15,** 1-8 (2014).

15. Mario, S. & Stephan, W.J.B. Gene prediction with a hidden Markov model and a new intron submodel. *Bioinformatics* **2003 Oct Suppl 2**, 215-225 (2003).

16. Initiative, T.A.G.J.N. Analysis of the genome sequence of the flowering plant. **408(6814)**, 796-815 (2000).

17. Blake, J.A., Christie, K.R., Dolan, M.E., Drabkin, H.J. & Westerfield, M. Gene Ontology Consortium: going forward. *Nucleic Acids Research* **43(D1),** D1049-D1056 (2015).

18. Mitchell A L, Attwood T K, Babbitt P C, et al. InterPro in 2019: improving coverage, classification and access to protein sequence annotations. *Nucleic Acids Research*, **47(D1),** D351-D360 (2019).

19. Emms, D.M. & Kelly, S. OrthoFinder: solving fundamental biases in whole genome comparisons dramatically improves orthogroup inference accuracy. *Genome biology* **16,** 1-14 (2015).

20. Guangchuang et al. ggtree: an r package for visualization and annotation of phylogenetic trees with their covariates and other associated data. *Methods in Ecology & Evolution* **8(1),** 28–36 (2017).

21. Love, M.I., Huber, W. & Anders, S. Moderated estimation of fold change and dispersion for RNA-seq data with DESeq2. *Genome Biology* **15,** 1-21 (2014).

22. Wang, Y. et al. MCScanX: a toolkit for detection and evolutionary analysis of gene synteny and collinearity. **40,** e49-e49 (2012).

23. Qiao, X. et al. Gene duplication and evolution in recurring polyploidization–diploidization cycles in plants. *Genome biology* **20,** 1-23 (2019).

24. Sudhir, K., Glen, S., Li, M., Christina, K. & Koichiro, T. MEGA X: Molecular evolutionary genetics analysis across computing platforms. *Molecular Biology & Evolution* **35(6),** 1547-1549 (2018).

25. Lim, J.Q. et al. BatMeth: improved mapper for bisulfite sequencing reads on DNA methylation. Genome Biol **13,** 1-14 (2012).
