# Supplementary material for: Chromosome-level genome assembly of Cornus officinalis reveals the evolution of loganin biosynthesis
Source: Hortic Res. 2025 Sep 24;13(1):uhaf259. doi: 10.1093/hr/uhaf259 (PMC12860560; doi:10.1093/hr/uhaf259)
Supplement: Web_Material_uhaf259 [file web_material_uhaf259.zip › Supplementary Tables 1-11.docx]

**Supplementary Tables**

**Table S1.** Summary statistics of *Cornus officinalis* assembly genome.

**Table S2.** The mapping rates of transcriptome to the assembled genome.

**Table S3.** BUSCO assessment for the genome assembly quality.

**Table S4.** Comparison of *Cornus officinalis* set with other species.

**Table S5.** Statistics of the repeat contents in the *Cornus officinalis* genome.

**Table S6.** The leaf RWC of 84K and NAC12-OE.

**Table S7.** Comparison of different types of transposons regions identified in eight plant genomes.

**Table S8.** Intact and solo-LTR elements were identified in three *Cornales* species.

**Table S9.** Enzyme Activity and Kinetic Characterization of three CoLAMTs.

**Table S10.** Loganin yield of CoLAMT in *Cornus officinalis.*

**Table S11.** Comparison of the two types of LAMT on the production of loganin.

**Table S1. Summary statistics of *Cornus officinalis* assembly genome**

| Type | Length (bp) |
| --- | --- |
| Length of anchored scaffolds | 481,066,559 |
| N50 of scaffolds (bp) | 297,711,837 |
| Total length of scaffolds (bp) | 3,017,603,859 |
| Numeber of scaffold | 366 |
| Numeber of contigs | 2,578 |
| Length of largest contig(bp) | 31,918,834 |
| Total length of contigs (bp) | 2,863,497,820 |
| N50 of contigs (bp) | 4,094,819 |
| GC content (%) | 39.13 |
| Total length of pseudomolecules (bp) | 3,017,603,859 |
| Sequences anchored to chromosomes (%) | 96.84 |
| Numeber of unanchored scaffolds (bp) | 95,289,278 |
| Total length of transposable elements (bp) | 2,077,439,870 |
| Percentage of transposable elements in genome size (%) | 72.55 |
| Number of gene models | 41,911 |
| Average transcript length (bp) | 8,145.78 |
| Average exons per number | 4.41 |
| Average exon length (bp) | 241.75 |
| Average CDS length (bp) | 1,066.06 |
| Number of annotated genes | 40,037 |
| Complete gene prediction BUSCO | 96.73 |

**Table S2. The mapping rates of transcriptome to the assembled genome**

| Tissues for RNA-seq | Overall mapping rate (%) | Properly mapped rate (%) |
| --- | --- | --- |
| old leaf | 94.81 | 92.78 |
| fresh leaf | 96.99 | 93.68 |
| seed | 96.59 | 93.42 |
| fruit | 97.82 | 93.35 |
| peel | 97.74 | 94.27 |
| Average | 96.79 | 93.5 |

**Table S3. BUSCO assessment for the genome assembly quality**

| Type | Number | Percentage (%) |
| --- | --- | --- |
| Complete BUSCOS(c) | 1,609 | 99.7 |
| Complete and single-copy | 1,533 | 94.98 |
| Complete and duplicated | 76 | 4.71 |
| Frafmented BUSCOs(F) | 3 | 0.19 |
| Missing BUSCOs(M) | 2 | 0.12 |
| Total BUSCO groups searched | 1,614 | 100 |

**Table S4. Comparison of *Cornus officinalis* set with other species**

| Species | Total number of genes | Average transcript length (bp) | Average CDS length (bp) | Average exon number per gene | Average exon length (bp) | Average intron length (bp) |
| --- | --- | --- | --- | --- | --- | --- |
| *Populus trichocarpa* | 36,350 | 5,258.30 | 957.25 | 3.65 | 262.56 | 1,625.56 |
| *Camellia sinensis* | 33,021 | 8,050.50 | 1,139.09 | 5.38 | 211.68 | 1,330.34 |
| *Camptotheca acuminata* | 51,735 | 8,394.99 | 1,656.63 | 6.14 | 269.81 | 1,052.31 |
| *Catharanthus rouseus* | 32,730 | 5,939.05 | 956.37 | 3.67 | 260.71 | 1,867.31 |
| *Cornus officinalis* | 41,911 | 8,145.78 | 1,066.06 | 4.41 | 241.75 | 2,076.33 |
| *Liriodendron chinense* | 35,269 | 10,589.50 | 1,266.42 | 4.89 | 258.9 | 2,395.79 |
| *Oryza sativa* | 42,183 | 2,369.36 | 1,108.28 | 4.21 | 263.26 | 392.88 |
| *Eucommia ulmoides* | 26,723 | 5,351.23 | 1,000.82 | 4.74 | 211.19 | 1,163.56 |
| *Arabidopsis thaliana* | 37,662 | 5,481.21 | 904.69 | 3.43 | 263.89 | 1,884.65 |

**Table S5. Statistics of the repeat contents in the *Cornus officinalis* genome**

|  |  | Type |  | Length (bp) | Proportion (%) |
| --- | --- | --- | --- | --- | --- |
| Class I Retrotransposon | | | |  |  |
| LTR | | | | 1,807,904,102 | 59.93 |
|  | | | Ty3/Gypsy | 1,245,019,957 | 41.26 |
|  |  |  | Ty1/Copia | 291,510,303 | 9.66 |
|  |  |  | BEL/Pao | 844,414 | 0.03 |
|  | | | Retroviral | 1,407,356 | 0.05 |
|  | | | others | 217,576,530 | 7.21 |
| LINE | | | | 49,704,973 | 1.65 |
|  | | | L1 | 44,183,266 | 1.46 |
|  |  |  | L2 | 5,521,707 | 0.21 |
| SINE | | | | 1,840,569 | 0.07 |
| Class II DNA transposon | | | |  |  |
|  | | | DNA transposons | 77,856,102 | 2.58 |
|  |  |  | hobo-Activator | 23,236,123 | 0.77 |
|  |  |  | Tc1-IS630-Pogo | 301,768 | 0.01 |
|  |  |  | Tourist/harbinger | 12,976,017 | 0.43 |
|  |  |  | others | 41,342,194 | 1.37 |
| Unclassified | | | | 378,011,349 | 12.53 |
| Total | | | | 2,263,771,553 | 75.04 |

**Table S6. The leaf RWC of 84K and NAC12-OE**

| Genotype | Condition | Leaf RWC (%) |
| --- | --- | --- |
| WT | Control | 90 |
| OE-2 | Control | 92 |
| OE-3 | Control | 91 |
| WT | Drought_28d | 65 |
| OE-2 | Drought_28d | 80 |
| OE-3 | Drought_28d | 82 |

**Table S7. Comparison of different types of transposons regions identified in eight plant genomes**

| Type | | *Cornus officinalis* | *Camptotheca acuminata* | *Camellia sinensis* | *Arabidopsis thaliana* | *Oryza sativa* | *Populus trichocarpa* | *Liriodendron chinense* | *Eucommia ulmoides* |
| --- | --- | --- | --- | --- | --- | --- | --- | --- | --- |
| Retroelements | | 59.93% | 19.89% | 36.07% | 8.16% | 25.07% | 21.76% | 56.94% | 39.86% |
| LTR elements | BEL/Pao | 0.03% | 0.01% | 0.46% | 0.01% | 0.00% | 0.00% | 0.02% | 0.04% |
|  | Retroviral | 0.05% | 0.03% | 1.07% | 0.00% | 0.00% | 0.02% | 0.01% | 0.03% |
|  | Gypsy/DIRS1 | 41.26% | 3.10% | 26.25% | 5.55% | 18.60% | 13.51% | 40.45% | 16.70% |
|  | Ty1/Copia | 9.66% | 11.34% | 6.22% | 1.59% | 4.83% | 6.49% | 13.08% | 13.22% |
|  | others | 7.21% | 0.94% | 0.68% | 0.16% | 0.35% | 0.38% | 1.67% | 7.23% |
| LINEs | | 1.65% | 4.01% | 1.36% | 0.78% | 1.12% | 0.86% | 1.69% | 2.52% |
| SINEs | | 0.07% | 0.46% | 0.03% | 0.07% | 0.17% | 0.50% | 0.02% | 0.12% |
| DNA transposons | | 2.58% | 3.33% | 3.84% | 3.27% | 14.77% | 6.24% | 5.80% | 4.22% |
| DNA | hobo-Activator | 0.77% | 1.85% | 1.55% | 0.40% | 1.24% | 1.45% | 3.53% | 1.42% |
|  | Tourist/Harbinger | 0.43% | 0.27% | 0.46% | 0.15% | 2.97% | 0.83% | 1.17% | 0.83% |
|  | Tc1-IS630-Pogo | 0.01% | 0.00% | 0.05% | 0.15% | 1.85% | 0.02% | 0.63% | 0.76% |
|  | others | 1.37% | 1.21% | 1.78% | 2.57% | 8.71% | 3.94% | 0.47% | 1.21% |
| Unclassified | | 12.53% | 13.98% | 26.89% | 1.13% | 1.26% | 7.05% | 1.04% | 17.16% |
| Total | | 75.04% | 37.20% | 66.80% | 12.56% | 41.09% | 35.05% | 63.78% | 61.24% |

**Table S8. Intact and solo-LTR elements were identified in three *Cornales* species**

| Species | Intact_LTR_Count | Solo_LTR_Count | Intact_to_Solo_LTR_Ratio |
| --- | --- | --- | --- |
| *C. officinalis* | 824 | 154 | 5.35 |
| *C. wilsoniana* | 583 | 254 | 2.3 |
| *C. controversa* | 437 | 207 | 2.1 |

**Table S9. Enzyme activity and kinetic characterization of three CoLAMTs**

| Enzyme | Km (μΜ) | Vmax (mol·mg-1·min^-1^) | kcat (s-1) | kcat/Km (M^-1^·s^-1^) | |
| --- | --- | --- | --- | --- | --- |
| Co1.482 | 13.62± 1.6 μM | 0.025 | 0.0394 | 2897.35 |  |
| Co1.482 | 13.58± 1.5 μM | 0.056 | 0.0427 | 3143.56 |  |
| Co1.482 | 9.85± 1.1 μM | 0.038 | 0.0404 | 4103.56 |  |
| Co507.206 | 83.41± 1.8 μM | 0.022 | 0.0246 | 294.93 |  |
| Co507.206 | 72.51± 2.3 μM | 0.035 | 0.0268 | 369.59 |  |
| Co507.206 | 48.83± 2.1 μM | 0.058 | 0.0193 | 395.29 |  |
| Co1486.99 | 94.21± 0.7 μM | 0.051 | 0.0266 | 282.37 |  |
| Co1486.99 | 114.66± 1.9 μM | 0.024 | 0.0269 | 234.61 |  |
| Co1486.99 | 96.16± 2.6 μM | 0.037 | 0.0251 | 261.58 |  |

**Table S10. Loganin yield of CoLAMT in *Cornus officinalis***

| OD600_of loganin strain | loganin_content | |
| --- | --- | --- |
| 0.2 | 2.12 3.23 2.64 | 3.18 3.52 3.71 |
| 0.4 | 5.32 7.76 4.51 | 6.03 5.17 4.93 |
| 0.6 | 8.39 7.97 9.43 | 9.03 7.62 8.84 |
| 0.8 | 7.54 6.58 5.36 | 8.30 6.17 4.53 |
| 1 | 0.23 0.18 0.13 | 0.12 0.07 0.26 |

**Table S11. Comparison of the two types of LAMT on the production of loganin**

| Enzymes | protein(ug) | Vol. of the reaction (ul) | Concentration of enzyme(mg/L) | Relative molecular weight(kD) | Kcat | Vmax | Km |
| --- | --- | --- | --- | --- | --- | --- | --- |
| CoLAMT | 28 | 100 | 280 | 85.492 | 60.76 | 0.038 | 0.0302± 1.5 |
| CaLAMT | 4 | 100 | 40 | 39.259 | 384.8 | 0.092 | 0.0948± 2.7 |
